# TRIM21 inhibits irradiation-induced mitochondrial DNA release and impairs antitumour immunity in nasopharyngeal carcinoma tumour models

**DOI:** 10.1038/s41467-023-36523-y

**Published:** 2023-02-16

**Authors:** Jun-Yan Li, Yin Zhao, Sha Gong, Miao-Miao Wang, Xu Liu, Qing-Mei He, Ying-Qin Li, Sheng-Yan Huang, Han Qiao, Xi-Rong Tan, Ming-Liang Ye, Xun-Hua Zhu, Shi-Wei He, Qian Li, Ye-Lin Liang, Kai-Lin Chen, Sai-Wei Huang, Qing-Jie Li, Jun Ma, Na Liu

**Affiliations:** 1 State Key Laboratory of Oncology in South China, Guangdong Key Laboratory of Nasopharyngeal Carcinoma Diagnosis and Therapy, Sun Yat-sen University Cancer Center, 510060 Guangzhou, P.R. China; 2 Collaborative Innovation Center for Cancer Personalized Medicine, Nanjing Medical University, Nanjing, China

**Keywords:** Radiotherapy, Head and neck cancer, Ubiquitylation, Tumour immunology

## Abstract

Although radiotherapy can promote antitumour immunity, the mechanisms underlying this phenomenon remain unclear. Here, we demonstrate that the expression of the E3 ubiquitin ligase, tumour cell-intrinsic tripartite motif-containing 21 (TRIM21) in tumours, is inversely associated with the response to radiation and CD8^+^ T cell-mediated antitumour immunity in nasopharyngeal carcinoma (NPC). Knockout of TRIM21 modulates the cGAS/STING cytosolic DNA sensing pathway, potentiates the antigen-presenting capacity of NPC cells, and activates cytotoxic T cell-mediated antitumour immunity in response to radiation. Mechanistically, TRIM21 promotes the degradation of the mitochondrial voltage-dependent anion-selective channel protein 2 (VDAC2) via K48-linked ubiquitination, which inhibits pore formation by VDAC2 oligomers for mitochondrial DNA (mtDNA) release, thereby inhibiting type-I interferon responses following radiation exposure. In patients with NPC, high TRIM21 expression was associated with poor prognosis and early tumour relapse after radiotherapy. Our findings reveal a critical role of TRIM21 in radiation-induced antitumour immunity, providing potential targets for improving the efficacy of radiotherapy in patients with NPC.

## Abstract (web summary)

The molecular mechanisms determining the response to radiotherapy remain incompletely understood. Here, the authors demonstrate that the E3 ubiquitin ligase and intracellular Fc receptor, TRIM21, impairs CD8^+^ T cell responses in nasopharyngeal carcinoma tumour models following ionizing radiation.

## Introduction

Radiotherapy is widely used to treat various types of solid tumours, and DNA damage in tumour cells is considered the main mechanism underlying its cytotoxic effect^1^. More recently, radiation-inducible innate and adaptive immune responses have emerged as an important research focus^2^. The expanded spectrum of neoantigens and enhanced antigen presentation in tumour cells^3^, as well as the recruitment and activation of dendritic cells (DCs) and CD8^+^ T cells^4^, induced by radiotherapy require the engagement of cytosolic DNA sensing and subsequent type-I interferon (IFN) signalling^5^. Activation of the cytoplasmic DNA sensing cyclic GMP–AMP synthase (cGAS)-stimulator of interferon genes (STING) pathway plays a predominant role in type-I IFN-mediated antitumour immunity^6,7^. However, tumour cell-intrinsic oncogenic pathways can facilitate the escape of tumour cells from this immune surveillance, resulting in tumour relapse.

The ubiquitin–proteasome system is a crucial homoeostatic regulator of protein degradation, function, and subcellular trafficking at the posttranslational level^8^. The dysfunction of E3 ubiquitin ligases is a key regulatory factor involved in the development and progression of various types of cancers^9^. Studies have noted that tumour-intrinsic E3 ligases mediate the regulation of programmed death ligand-1 (PD-L1) expression^10^ and macrophage infiltration^11^, highlighting the role of the ubiquitin system in antitumour immunity. Tripartite motif-containing 21 (TRIM21) is a member of the TRIM protein family of RING E3 ubiquitin ligases^12^. It was first identified as an antibody-binding protein in innate immune cells and acts as a cytosolic Fc receptor to control viral infection^13^. TRIM21 promotes cancer progression by destabilising tumour suppressor proteins, such as p53 and p27^14^. Other studies have reported that TRIM21 is involved in the mTOR cascade^15^, aerobic glycolysis^16^ and amino acid metabolism^17^ in cancer cells. However, whether TRIM21 expression in tumours affects antitumour immunity, especially that induced by radiotherapy, is largely unknown.

Here, based on analysis of bulk and single-cell RNA sequencing (scRNA-seq) data, we first show that the expression of the E3 ligase TRIM21 is inversely associated with radiation-induced antitumour immunity in nasopharyngeal carcinoma (NPC). Moreover, TRIM21 expression in tumour cells facilitates the ubiquitination and degradation of voltage-dependent anion-selective channel protein 2 (VDAC2) and impedes pore formation by VDAC2 oligomers for mitochondrial DNA (mtDNA) release, thus suppressing radiation-induced STING–type-I IFN signalling. In addition, we reveal the clinical significance of higher TRIM21 expression in NPC patients and provide proof-of-concept evidence that targeting TRIM21 with genetic tools may enhance the antitumour efficacy of radiotherapy.

## Results

### Tumour-intrinsic TRIM21 correlates inversely with CD8^+^ T-cell antitumour immunity

**Fig. 1:**
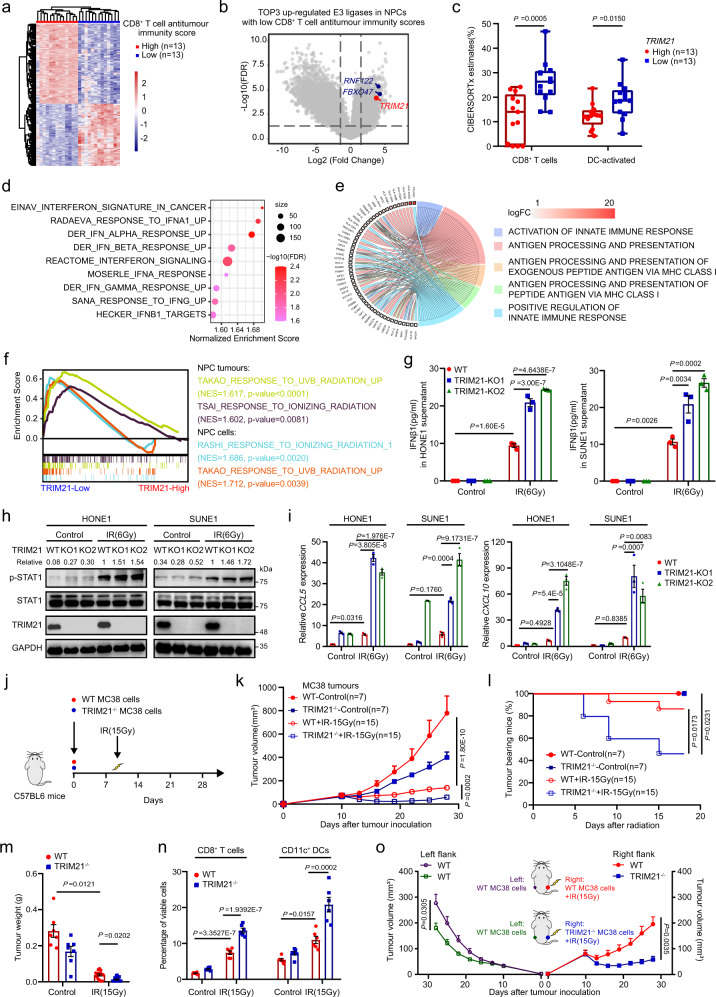
TRIM21 expression in tumour cells impairs radiation-induced T-cell-mediated antitumour immunity. **a** Heatmap showing the clustering of NPC tumours with high or low CD8^+^ T-cell-mediated antitumour immunity scores. **b** Volcano plot showing that three E3 ligases, *RNF122*, *FBXO47* and *TRIM21*, were upregulated in NPC tumours with low CD8^+^ T-cell-mediated antitumour immunity scores. **c** CIBERSORTx analysis of CD8^+^ T cells and activated DCs that infiltrated into tumours with high or low *TRIM21* expression (two-tailed unpaired *t* test). **d** GSEA suggesting impaired type-I IFN responses in tumours with high *TRIM21* expression. **e** GO analysis of the scRNA-seq showing a negative association between *TRIM21* expression and the ‘activation of innate immune response’ and ‘antigen processing and presentation’ terms in NPC cells. **f** GSEA showed a poor radiation response in *TRIM21*-high tumours and cells (two-tailed unpaired *t* test). **g**–**i** WT and TRIM21-KO NPC cells were treated with or without IR. After 48 h, IFN-β1 concentration in the culture supernatant (**g**, ELISA), pSTAT1 levels (**h**, western blot analysis), and *CCL5* and *CXCL10* expression levels (**i**, qPCR) were measured (one-way ANOVA with the Tukey’s multiple comparisons test). **j**–**n** In total, 5 × 10^5^ WT or TRIM21^−/−^ MC38 cells were inoculated into C57BL/6 mice. Mice in the IR-treated groups were subjected to focal radiation with a single fraction of 15 Gy on day 10 after tumour cell inoculation (**j**). The tumour growth rate (**k**, two-way ANOVA), tumour eradication rate (**l**, log-rank test), weight of excised residual tumours (**m**, *n* = 7 in each control group, *n* = 15 in each IR group, Kruskal–Wallis test with Dunn’s multiple comparisons test), and proportions of tumour-infiltrating CD8^+^ T cells and CD11c^+^ DCs (**n**, *n* = 7 in each control group, *n* = 6 in each IR group, one-way ANOVA with Tukey’s test for multiple comparisons) are reported. **o**, Tumour growth of the unirradiated abscopal tumours (WT-MC38, left flank) and the irradiated (15 Gy) primary tumours (WT-MC38 or TRIM21^−/−^ MC38, right flank) in C57/BL6 mice (*n* = 9 in each group, two-way ANOVA). The data are shown as the mean ± SEM and are representative of three independent experiments (**g**–**i**).

To identify potential oncogenic targets that may impair T-cell-mediated antitumour immunity, we first characterised 128 NPC patients^18^ with weak or robust T-cell antitumour immunity using a score that takes into account the expression of gene sets previously reported to be indicators of elevated CD8^+^ T-cell infiltration and antitumour responses^19^. Survival analysis showed that the patients with low T-cell-mediated antitumour immunity scores had poor disease-free survival after radical treatment (Supplementary Fig. 1a), supporting that the abundance of tumour lymphocyte infiltration can affect treatment efficacy^20^. As expected, tumours with weak and robust T-cell-mediated antitumour immunity scores exhibited distinct gene expression profiles (Fig. 1a), and three E3 ubiquitin ligases were markedly upregulated in tumours from patients with weak T-cell-mediated antitumour immunity scores (Fig. 1b). As only TRIM21 was significantly associated with poor disease-free survival in NPC and its expression level was negatively correlated with the transcript levels of genes associated with CD8^+^ T-cell infiltration and antitumour responses, such as *CD8A*, *PRF1*, *GZMA*, *GZMB*, *IFNG* and *LCK* (Supplementary Fig. 1b, c), we selected TRIM21 for further study. CIBERSORTx analysis further confirmed that NPC tumours with higher TRIM21 expression contained fewer infiltrated CD8^+^ T cells and activated DCs (Fig. 1c), suggesting that TRIM21 may play a vital role in suppressing T-cell-mediated antitumour immunity.

### TRIM21 inhibits radiation-induced type-I IFN signalling and T-cell antitumour immunity

To elucidate the effect of TRIM21 on T-cell-mediated antitumour immunity, we performed gene set enrichment analysis (GSEA) and demonstrated that TRIM21-high tumours showed weak IFN responses, especially the type-I IFN response (Fig. 1d). As TRIM21 has been shown to negatively regulate the type-I IFN response in myeloid DCs^21^, we conducted Gene Ontology (GO) term enrichment analysis on our scRNA-seq data^18^ to investigate the function of TRIM21 in tumour cells. High TRIM21 expression in NPC cells was negatively related to activation of the innate immune response and to antigen processing and presentation (Fig. 1e). Notably, in both tumours and cells, high TRIM21 expression was correlated with a poorer response to ionising radiation (IR) (Fig. 1f). Our findings suggest that tumour-intrinsic TRIM21 may function as a negative regulator of radiotherapy-inducible type-I IFN activation-mediated antitumour immunity.

To verify this hypothesis, we deleted the *TRIM21* gene in HONE1 and SUNE1 cells by CRISPR/Cas9 gene editing (Supplementary Fig. 1d) and exposed these cells to IR. As expected, IFN-β1 expression was significantly upregulated at both the mRNA and protein levels upon IR treatment and was notably enhanced after TRIM21 knockout (KO) (Fig. 1g and Supplementary Fig. 1e). Consistent with the activation of type-I IFNs, increased phosphorylation of STAT1 was found after IR, especially in TRIM21-KO cells (Fig. 1h). Considering the effect of TRIM21 on lymphocyte infiltration (Fig. 1c), we analysed the correlations between the expression of TRIM21 and that of chemokines that control DC and T-cell recruitment in the scRNA-seq data and found marked negative correlations between TRIM21 and the IFN-stimulated genes (ISGs) *CCL5* and *CXCL10* (Supplementary Fig. 1f). Consistent with the above results, depletion of TRIM21 prominently enhanced the expression of *CCL5* and *CXCL10* after IR treatment (Fig. 1i).

To explore whether TRIM21 mediates antitumour immunity, we employed the MC38 mouse model, as TRIM21 knockout also led to higher *Ifnb1* expression but it had no obvious effect on the radiosensitivity of MC38 cells (Supplementary Fig. 1g–i). We implanted TRIM21 wild-type (WT) or knockout MC38 cells into C57BL/6 mice and then monitored tumour growth after IR treatment (Fig. 1j). Compared to TRIM21-WT tumours, TRIM21-KO tumours displayed obvious growth retardation following IR treatment (Fig. 1k). More than half of the TRIM21-KO tumours (8/15, 53.3%) completely regressed, and none recurred (Fig. 1l). In addition, the weight of residual TRIM21-KO tumours was markedly lower than that of TRIM21-WT tumours (Fig. 1m). Moreover, flow cytometric analysis showed that TRIM21 deficiency resulted in elevated infiltration of CD8^+^ T cells and CD11c^+^ DCs, as well as CD45^+^ immune cells and CD3^+^ T cells (Fig. 1n and Supplementary Fig. 1j). In addition, the proportions of CD69^+^ or effector molecule granzyme B (GZMB)^+^ cells among tumour-infiltrating CD8^+^ T cells were higher in irradiated TRIM21-KO tumours (Supplementary Fig. 1k), indicating the IR-induced activation of the CD8^+^ T-cell antitumour immune response in TRIM21-KO tumours. Furthermore, both depletion of CD8^+^ T cells and IFNAR1 blockade abolished the growth retardation of TRIM21-deficient tumours after radiation treatment (Supplementary Fig. 1l, m).

As radiotherapy can result in antitumour immune response-mediated abscopal effects, i.e., that treatment of primary tumours with radiation leads to the regression of distant secondary tumours not treated with radiation^22^, we then tested whether TRIM21 deficiency potentiates the abscopal effects of radiotherapy. Surprisingly, we observed a significant growth delay of abscopal tumours in mice bearing primary TRIM21-deficient tumours after radiation treatment compared to the abscopal tumours in mice bearing primary WT tumours (Fig. 1o). Collectively, these findings indicate that TRIM21 impairs CD8^+^ T-cell antitumour immunity and radiation efficacy by inhibiting the activation of radiation-induced type-I IFN signalling.

### TRIM21 deficiency facilitates cytosolic mtDNA release to activate STING signalling

**Fig. 2:**
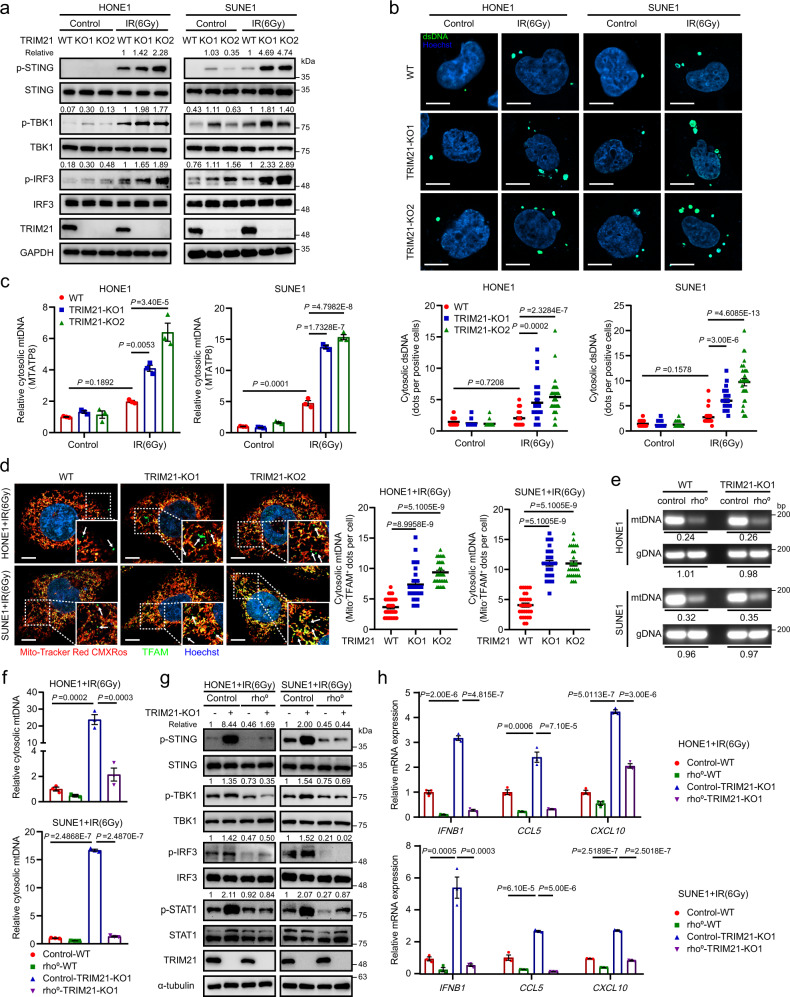
TRIM21 deficiency promotes cytosolic mtDNA release to activate STING signalling. **a**–**c** WT and TRIM21-KO NPC cells were treated with or without IR. **a** After 48 h, the levels of total and phosphorylated STING, TBK1 and IRF3 were measured by western blot analysis. **b** Cytosolic DNA accumulation was assessed by IF staining with a dsDNA-specific antibody 24 h after IR. Representative images (scale bar, 10 μm) and quantitative results are shown (*n* = 30 cells per group). **c** The abundance of *MTATP8* DNA sequence in the cytosolic fraction was measured by qPCR 24 h after IR. **d** Representative images showing cytosolic mtDNA accumulation in irradiated WT and TRIM21-KO NPC cells 24 h after IR treatment (scale bar, 10 μm). TFAM (mitochondrial transcription factor A, green) not associated with mitochondria (MitoTracker Red CMXRos) was considered as cytosolic mtDNA (as indicated by the white arrows). Quantitative results from 30 cells per group are reported. **e** The amounts of gDNA (*GAPDH*) and mtDNA *(MTATP8*) in control and rho^0^ NPC cells were estimated by qPCR. Rho^0^/control ratios are reported. **f** *MTATP8* abundance in the cytosolic fraction of control and rho^0^ NPC cells 24 h after IR treatment. **g**, **h** Control and rho^0^ NPC cells were treated with IR. After 48 h, the levels of phosphorylated STING, TBK1, IRF3, and STAT1 (**g**) and the relative expression levels of *IFNB1*, *CCL5* and *CXCL10* were measured. The results are representative of three independent experiments (**a**–**h**). The data are presented as the mean ± SEM; one-way ANOVA with Tukey’s test for multiple comparisons (**b**–**d**, **f**, and **h)**.

Activation of the STING/TBK1/IRF3 axis is directly responsible for type-I IFN response during radiation^2^. We therefore examined the effect of TRIM21 depletion on IR-inducible activation of STING signalling and found that TRIM21 depletion significantly promoted IR-induced phosphorylation of STING, TBK1 and IRF3 (Fig. 2a). In addition, knockdown of STING abolished TRIM21 knockout-mediated activation of the STING pathway and the enhanced expression of *IFNB1* and ISGs after IR treatment (Supplementary Fig. 2a, b), indicating function of TRIM21further upstream in the STING signalling pathway.

As cGAS functions as a major sensor of double-stranded DNA (dsDNA) and is responsible for STING activation^6,23^, we sought to determine whether TRIM21 directly targets cGAS. Interestingly, TRIM21 depletion did not change the expression of cGAS upon IR treatment (Supplementary Fig. 2c). In addition, IFI16 is reported as a TRIM21 downstream and DNA sensor to activate STING-dependent type-I IFN signalling^24^, we thus investigated the interaction between TRIM21 and IFI16 in NPC cells, and surprisingly found that TRIM21 did not interact with IFI16 (Supplementary Fig. 2d). We therefore reasoned that TRIM21 might influence the accumulation of cytosolic dsDNA in irradiated cells. High-resolution confocal microscopy and quantitative image analysis showed that TRIM21 knockout resulted in an increased abundance of cytosolic dsDNA in NPC cells after IR treatment (Fig. 2b). As genomic DNA (gDNA) is regarded as the main source of cytosolic dsDNA during IR exposure^25^, we first detected cytosolic gDNA by detecting the glyceraldehyde-3-phosphate dehydrogenase (GAPDH) DNA sequence in NPC cells treated with IR. The results showed that the cytosolic gDNA accumulation was increased in the irradiated cell, while the TRIM21 knockout did not remarkably affect the cytosolic gDNA accumulation induced by DNA damage (Supplementary Fig. 2e, f). Recent studies have highlighted the activating role of mtDNA in radiation-induced immune activation^26,27^, we thus further evaluated the enrichment of cytosolic mtDNA by using qRT-PCR to assess the relative mitochondrially encoded ATP synthase 8 (*MTATP8*, a mtDNA encoded gene) abundance^26^ and by conducting immunofluorescence (IF) experiment through staining with the MitoTracker Red CMXRos (a mitochondrial tracer dye) and the antibody against mitochondrial transcription factor A (TFAM)^28^. The results revealed an increased level of *MTATP8* in the cytosolic fraction (Fig. 2c), as well as accumulation of TFAM in the cytosol of irradiated TRIM21-KO cells (Fig. 2d).

To explore whether the enrichment of cytosolic mtDNA is a critical event in TRIM21 knockout-induced enhancement of type-I IFN responses, we generated mtDNA-depleted (rho^0^) TRIM21-WT and TRIM21-KO NPC cells (Fig. 2e) and found that the abundance of cytosolic dsDNA (Supplementary Fig. 2g) and mtDNA (Fig. 2f) was dramatically decreased in rho^0^ cells either with or without TRIM21 depletion after IR treatment. Moreover, mtDNA depletion completely abolished the activation of STING–type-I IFN signalling and increased in the expression of *IFNB1* and ISGs in TRIM21-KO cells (Fig. 2g, h). Our findings thus indicate that TRIM21 depletion enhances radiation-induced mtDNA release to activate STING signalling.

### TRIM21 promotes VDAC2 degradation by increasing its K48-linked ubiquitination

**Fig. 3:**
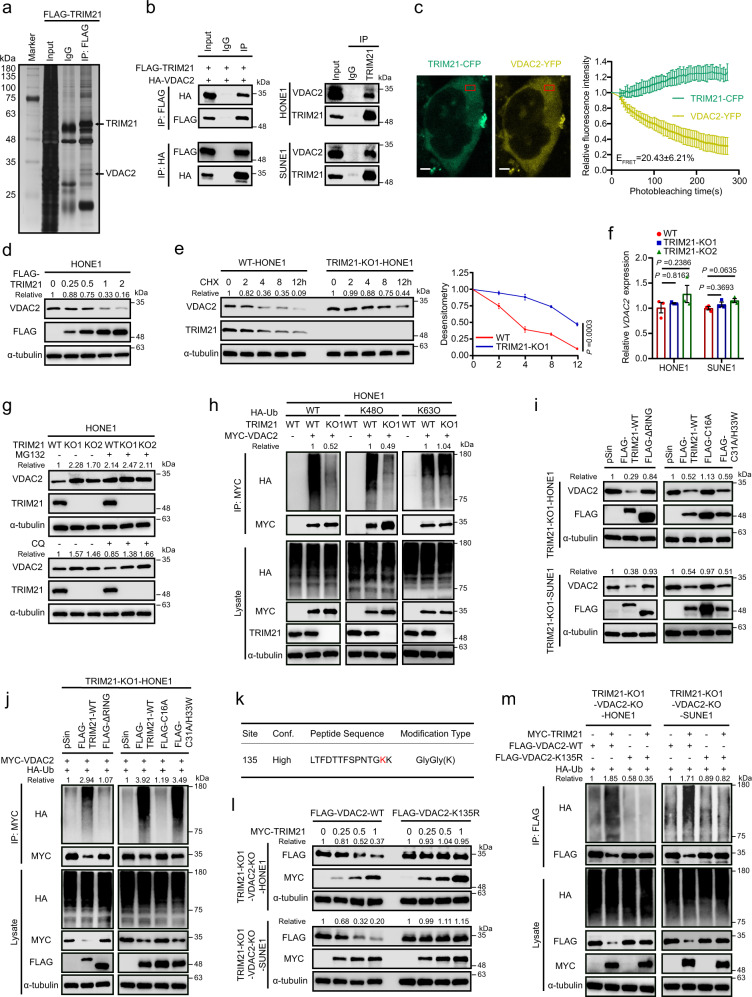
TRIM21 promotes the degradation of VDAC2 by increasing its K48-linked ubiquitination. **a** Silver staining of SDS–PAGE gels showed that the FLAG immunoprecipitates were pulled down from HONE1 cells overexpressing FLAG-TRIM21. The proteins of interest are indicated. **b** Left: Co-IP with an anti-FLAG (top) or an anti-HA (bottom) antibody revealed the interaction of exogenous TRIM21 and VDAC2 in HEK293T cells. Right: Co-IP with an anti-TRIM21 antibody in NPC cells revealed the interaction of endogenous TRIM21 and VDAC2. **c** FRET assay in HEK293T cells transfected with TRIM21-CFP and VDAC2-YFP. Representative cells with TRIM21-CFP and VDAC-YFP, and region of FRET analysis was indicated. The exponential decrease in VDAC2-YFP (acceptor) fluorescence and the concomitant increase in TRIM21-CFP (donor) fluorescence during YFP-photobleaching were analysed in 20 cells. The FRET efficacy (E_FRET_) was reported. Scale bar, 5 μm. **d** VDAC2 protein level in HONE1 cells transfected with gradient concentrations of FLAG-tagged *TRIM21*. **e** Immunoblots (left) and corresponding greyscale analysis (right) of VDAC2 in WT and *TRIM21*-KO HONE1 cells treated with CHX for the indicated times. **f** *VDAC2* mRNA expression in TRIM21-KO NPC cells. **g** VDAC2 protein level in WT and TRIM21-KO HONE1 cells after 6 h of treatment with MG132 (top) or CQ (bottom). **h** Ubiquitination level of exogenous MYC-VDAC2 in WT and TRIM21-KO HONE1 cells. **i** VDAC2 protein level in TRIM21-KO NPC cells transfected with vector, FLAG-tagged WT *TRIM21* or three FLAG-tagged *TRIM21* mutants. **j** The ubiquitination of MYC-tagged VDAC2 in *TRIM21*-KO HONE1 cells transfected with vector, FLAG-tagged WT *TRIM21* or three FLAG-tagged *TRIM21* mutants. **k** MS analysis of VDAC2 ubiquitination sites. **l** Protein levels of FLAG-tagged WT and K135R VDAC2 in NPC cells transfected with gradient concentrations of MYC-tagged *TRIM21*. **m** Ubiquitination of FLAG-tagged WT and K135R VDAC2 by MYC-tagged TRIM21. The results are representative of three independent experiments (**a**–**j**, **l**, **m**). The data are presented as the mean ± SEM (**c**, **e**). Comparisons were performed using two-way ANOVA (**e**) and one-way ANOVA with Tukey’s test for multiple comparisons (**f**).

The molecular mechanisms by which TRIM21 impairs cytosolic mtDNA accumulation remain unknown. A reasonable assumption is that TRIM21 targets the key regulators of mitochondrial permeability, such as BAX, which has been reported to facilitate IR-induced mtDNA efflux^26^. However, the co-IP assays showed there was no interaction between TRIM21 and BAX (Supplementary Fig. 3a). We thus conducted mass spectrometry (MS) to determine the potential targets of TRIM21 and identified VDAC2, an important channel protein that belongs to VDAC family including VDAC1/2/3 proteins and has been previous reported to connect mitochondrial and cytosolic compartments^29^ (Fig. 3a, Supplementary Fig. 3b and Supplementary Table 1). Coimmunoprecipitation (Co-IP) analysis verified the exogenous and endogenous interactions between TRIM21 and VDAC2 proteins (Fig. 3b), and these interactions were confirmed by the colocalization of these two proteins in the cytoplasm, as visualised by immunofluorescence (IF) staining (Supplementary Fig. 3c). As TRIM21 is reported as a cytosolic Fc receptor that can bind antibodies^30^ and result in a misleading interaction in Co-IP assay, we further conducted fluorescence energy resonance transfer (FERT) assay^31^ to confirm the interaction between TRIM21 and VDAC2. We observed a concomitant increase (about 20.43 ± 6.21%) in TRIM21-CFP fluorescence when the VDAC2-YFP fluorescence was iteratively photobleached, indicating a strong interaction between TRIM21 and VDAC2 (Fig. 3c and Supplementary Fig 3d). And TRIM21 had no interactions with the VDAC1 or VDAC3 (Supplementary Fig. 3e). To map the binding sites in TRIM21 and VDAC2, we analysed the interaction between HA-tagged VDAC2 and Flag-tagged full-length TRIM21 and its truncation mutants. The lack of the carboxy-terminal SPRY and PRY domains prominently impaired the interaction between TRIM21 and VDAC2 (Supplementary Fig. 3f), indicating that these domains are required for the interaction between TRIM21 and VDAC2.

The regulation of TRIM21 on VDAC2 was next investigated. We found the TRIM21 overexpression decreased the protein level of endogenous VDAC2 in a dose-dependent manner (Fig. 3d and Supplementary Fig. 3g). Conversely, knockout of TRIM21 dramatically inhibited VDAC2 degradation after cycloheximide (CHX) treatment, suggesting that TRIM21 could shorten the half-life of the VDAC2 protein (Fig. 3e and Supplementary Fig. 3h). In addition, neither knockout nor overexpression of TRIM21 affected the VDAC2 mRNA level (Fig. 3f and Supplementary Fig. 3i**)**. Considering that E3 ligases were usually promoted the degradation of their substrates through ubiquitin–proteasome pathway or the autophagy–lysosome pathway^32^, we treated TRIM21-WT and TRIM21-KO cells with MG132 (a proteasome inhibitor) or chloroquine (CQ; an autophagy inhibitor) and revealed that the TRIM21-mediated VDAC2 degradation was reversed by MG132 but not CQ, indicating that TRIM21 degrades the VDAC2 protein through the ubiquitin–proteasome pathway (Fig. 3g and Supplementary Fig. 4a). Consistent with this finding, knockout of TRIM21 significantly decreased the K48-linked but not the K63-linked polyubiquitination of VDAC2 (Fig. 3h and Supplementary Fig. 4b).

To clarify the ubiquitinase active domain and site of TRIM21 for VADC2 ubiquitination, we constructed three previously reported “RING mutants” of TRIM21: a RING domain deletion (∆RING) mutant, C16A mutant and C31A/H33W mutant^12,33^. We found that neither the ∆RING mutant nor the C16A mutant could degrade VDAC2 (Fig. 3i). Consistent with this finding, restoring TRIM21 expression in TRIM21-KO NPC cells with TRIM21-WT or the C31A/H33W mutant rescued the ubiquitination of VDAC2, while restoration with the ∆RING or C16A mutant did not (Fig. 3j and Supplementary Fig. 4c), demonstrating that the RING domain is required for the E3 ligase activity of TRIM21 and that the C16 site in the RING domain of TRIM21 is responsible for the ubiquitination of VDAC2.

To search for the ubiquitinated sites in VDAC2, we performed MS with HONE1 cells co-transfected with HA-tagged ubiquitin (HA-Ub) and MYC-tagged VDAC2 **(**MYC-VDAC2; Fig. 3k, Supplementary Fig. 4d and Supplementary Table 2). Then, a Lys/Arg substitution mutant (K135R) of VDAC2 was generated. By restoring the expression of TRIM21 and VDAC2 (WT or the K135R mutant) in TRIM21/VDAC2 double-knockout NPC cells, we found that the half-life of VDAC2-K135R was significantly prolonged compared to that of VDAC2-WT (Fig. 3l). Consistent with this finding, VDAC2-K135R was resistant to TRIM21-mediated ubiquitination (Fig. 3m). Taken together, our findings demonstrate that TRIM21 mediates the K48-linked ubiquitination of VDAC2 at K135.

### TRIM21 depletion facilitates VDAC2 oligomerization-mediated cytosolic mtDNA release

**Fig. 4:**
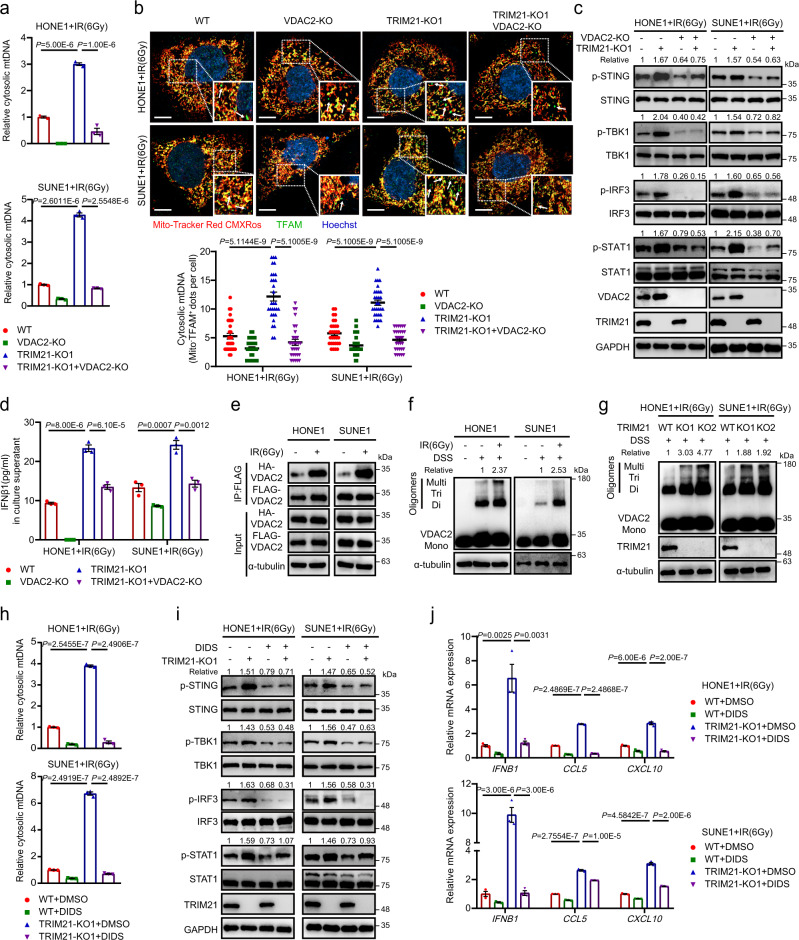
TRIM21 depletion facilitates VDAC2 oligomerization-mediated mtDNA release. **a**–**d** The indicated NPC cells were treated with IR. The relative *MTATP8* abundance in the cytosolic fractions was assessed by qPCR (**a**). Cytosolic mtDNA accumulation was evaluated by quantification of cytoplasmic TFAM (indicated by the white arrows) 24 h after IR treatment. Representative images (scale bar, 10 μm) and quantitative results are shown (*n* = 30 cells per group) (**b**). The levels of total and phosphorylated STING, TBK1, IRF3 and STAT1 (**c**) and the IFN-β1 level in culture supernatants (**d**) were measured 48 h after IR treatment. **e** Co-IP with an anti-FLAG antibody revealed the enhanced interaction of two exogenous VDAC2 monomers in lysates of irradiated NPC cells. **f** Immunoblot analysis showed an increased level of VDAC2 oligomers in NPC cells 24 h after IR treatment. **g** VDAC2 oligomers in WT and TRIM21-KO NPC cells 24 h after IR treatment. **h**–**j** NPC cells were pre-treated with the VDAC2 oligomerization inhibitor DIDS (100 μM) or DMSO for 24 h and were then treated with IR. The relative cytosolic *MTATP8* abundance was assessed by qPCR 24 h after IR treatment (**h**). The levels of phosphorylated STING, TBK1, IRF3 and STAT1 (**i**), as well as the expression levels of *IFNB1*, *CCL5*, and *CXCL10* (**j**), were measured 48 h after IR treatment. *n* = 3 independent experiments. The data are presented as the mean ± SEM (**a**, **b**, **d**, **h** and **j**). Comparisons were performed using one-way ANOVA with the Tukey’s test for multiple comparisons.

We next investigated the mechanism by which VDAC2 affects the release and accumulation of cytosolic mtDNA in NPC cells during IR treatment and revealed that knockout of VDAC2 significantly decreased the abundance of cytosolic mtDNA fragments in irradiated TRIM21-WT and TRIM21-KO NPC cells (Fig. 4a). Moreover, knockout of VDAC2 markedly reversed the TRIM21 depletion-mediated increase in TFAM accumulation in the cytoplasm of NPC cells (Fig. 4b). After the reduction in cytosolic mtDNA in TRIM21/VDAC2 double-knockout cells, the IR-induced activation of STING and type-I IFN signalling, the concentration of IFN-β1 in the culture supernatant, and the mRNA expression levels of *IFNB1* and ISGs were notably reduced compared to those in TRIM21-KO cells (Fig. 4c, d and Supplementary Fig. 5a).

VDAC2 has been reported to play a critical role in regulating the oligomerization of BAK and BAX in apoptotic cells^34,35^. Thus, we first investigated whether mtDNA release in irradiated NPC cells is facilitated by BAX/BAK macropores, as previously reported^26^. Unfortunately, treatment with BAI1^36^, a BAX oligomerization inhibitor, did not reduce the expression of *IFNB1* and ISGs upon IR treatment, indicating that IR-induced mtDNA release in NPC cells occurs in a BAK/BAX-independent manner (Supplementary Fig. 5b). Recent studies report that mtDNA releases through mitochondrial permeability transition pore (mPTP) in the inner mitochondrial membrane and VDAC1/3 oligomers in mitochondrial outer membrane^37,38^. Although the mPTP opening through visualising the decreased Calcein fluorescence was induced upon IR treatment, knockout of TRIM21 did not affect the mPTP opening in irradiated NPC cells (Supplementary Fig. 5c). We next investigated whether VDAC1/3 oligomers are responsible for the mtDNA-induced type-I IFN responses in irradiated NPC cells. However, neither knockout of VDAC1 nor the knockdown of VDAC3 could influence the expression of *IFNB1* and ISGs upon IR treatment (Supplementary Fig. 5d–g).

Since VDAC2 can oligomerize under mROS stimulation^39^, we hypothesised that VDAC2 directly facilitates mtDNA release by oligomerizing to form mitochondrial pores upon IR exposure. As expected, we found an interaction between VDAC2 monomers, which was markedly strengthened after IR treatment (Fig. 4e and Supplementary Fig. 5h). Moreover, the formation of VDAC2 trimers and higher-order oligomers was observed in NPC cells upon IR treatment (Fig. 4f) and was enhanced after TRIM21 knockout (Fig. 4g). Treatment with DIDS, a VDAC oligomerization inhibitor^40^, dramatically diminished the accumulation of cytosolic mtDNA in TRIM21-KO cells after IR treatment by detecting cytosolic *MTATP8* amount with qRT-PCR (Fig. 4h) and TFAM accumulation with IF assays (Supplementary Fig. 5i). Consistent with this finding, the activation of STING and type-I IFN signalling, as well as the increases in *IFNB1* and ISG expression mediated by TRIM21 depletion, was reduced after DIDS treatment (Fig. 4j, k). Overall, these results support the hypothesis that TRIM21 depletion facilitates radiation-induced VDAC2 oligomerization to release cytosolic mtDNA.

### TRIM21 deficiency potentiates radiation-induced antigen presentation

**Fig. 5:**
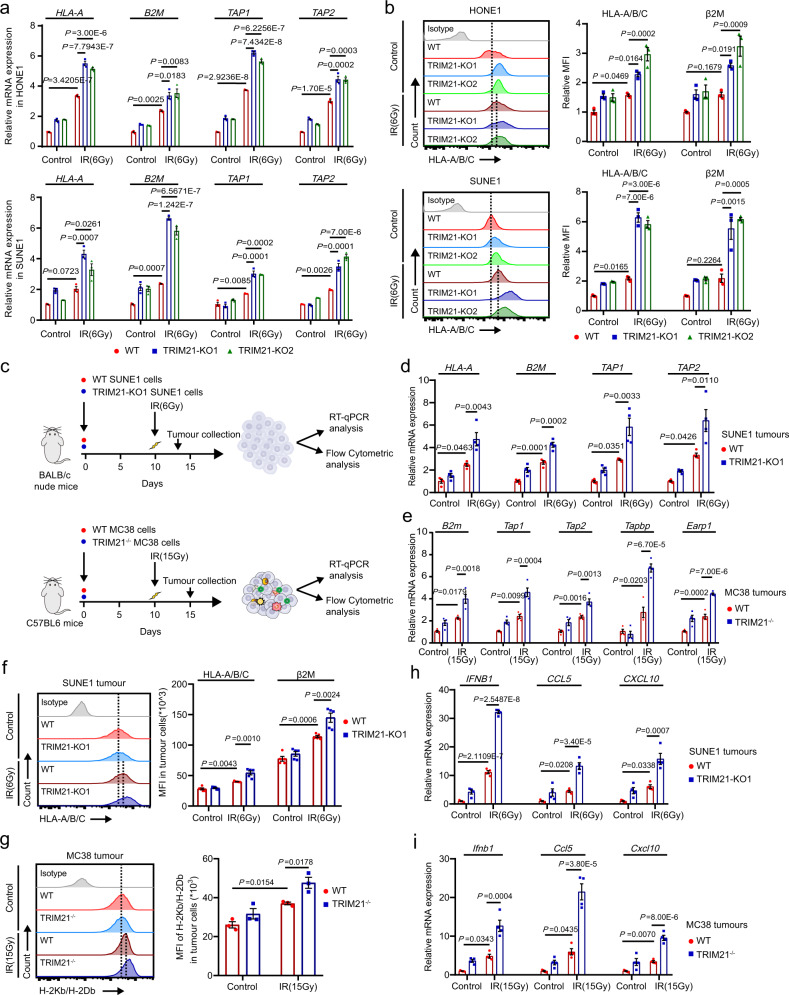
TRIM21 deficiency potentiates radiation-induced antigen presentation. **a**, **b** WT and TRIM21-KO NPC cells were treated with or without IR. After 48 h, the mRNA expression of antigen presentation molecules, including *HLA-A*, *B2M*, *TAP1* and *TAP2*, was measured by qPCR (**a**), and the surface MHC-I molecules HLA-A/B/C and β2m were analysed by flow cytometry (**b**). The results are representative of three independent experiments. **c** A total of 1 × 10^6^ WT or TRIM21-KO SUNE1 cells were subcutaneously injected into the flanks of BALB/c nude mice, and 1 × 10^6^ WT or TRIM21^−/−^ MC38 cells were subcutaneously injected into the flanks of C57BL/6 mice. Tumour formation was confirmed 10 days later, and the SUNE1 and MC38 tumours in mice in the IR-treated groups were then subjected to focal radiation with a single fraction of 6 Gy or 15 Gy, respectively. Tumours were dissected 3 or 5 days after IR. The expression of antigen presentation molecules, including *HLA-A*, *B2M*, *TAP1* and *TAP2* in SUNE1 tumours (**d**, *n* = 4) and *B2m*, *Erap1*, *Tapbp*, *Tap1* and *Tap2* in MC38 tumours (**e**, *n* = 4), was measured by RT–qPCR, and surface MHC-I molecules on murine CD45^–^ cells in SUNE1 tumours (**f**, *n* = 5) and MC38 tumours (**g**, *n* = 3) were analysed by flow cytometry. **h**, **i** qPCR analysis of *IFNB1*, *CCL5* and *CXCL10* mRNA expression in SUNE1 tumours (**h**, *n* = 4) and MC38 tumours (**i**, *n* = 4) as indicated in (**c**). All data are presented as the mean ± SEM. Statistical analysis was performed by one-way ANOVA with Tukey’s test for multiple comparisons (**a**, **b** and **d**–**i**).

We next investigated the mechanism by which TRIM21 affects the IR-induced antitumour immune response. We found that TRIM21 knockout significantly increased the expression of the antigen presentation molecules *HLA-A*, *B2m*, *TAP1*, and *TAP2* in IR-treated cells (Fig. 5a). Consistent with this finding, flow cytometric analysis confirmed the upregulation of HLA-A/B/C and β2M on the surface of TRIM21-KO NPC cells after IR treatment (Fig. 5b). Knockout of VDAC2 in NPC cells dramatically reversed the TRIM21 deficiency-induced increases in the expression levels of antigen presentation molecules upon IR treatment (Supplementary Fig. 6a, b). These data show that TRIM21 deficiency enhances IR-induced antigen presentation by increasing the protein level of VDAC2 in NPC cells.

We further validated the in vivo function of TRIM21 in antigen presentation (Fig. 5c). Knockout of TRIM21 resulted in high expression of *HLA-A*, *B2M*, *TAP1* and *TAP2* in SUNE1 tumour cells isolated from BALB/c nude mice (Fig. 5d) and of *B2m*, *Tap1, Tap2, Tapbp* and *Earp1* in MC38 tumour cells isolated from C57BL/6 mice (Fig. 5e). Subsequent flow cytometric analysis confirmed that the expression of surface MHC class I molecules on SUNE1 and MC38 cells was significantly increased in TRIM21-KO tumours after IR treatment (Fig. 5f, g). In addition, the mRNA expression of *IFNB1*, *CCL5* and *CXCL10* was markedly increased in TRIM21-KO tumours after IR treatment (Fig. 5h, i). Collectively, these findings demonstrate that TRIM21 deficiency in tumour cells results in enhanced IR-induced antigen presentation.

### TRIM21 depletion enhances radiation-induced antitumour immunity

**Fig. 6:**
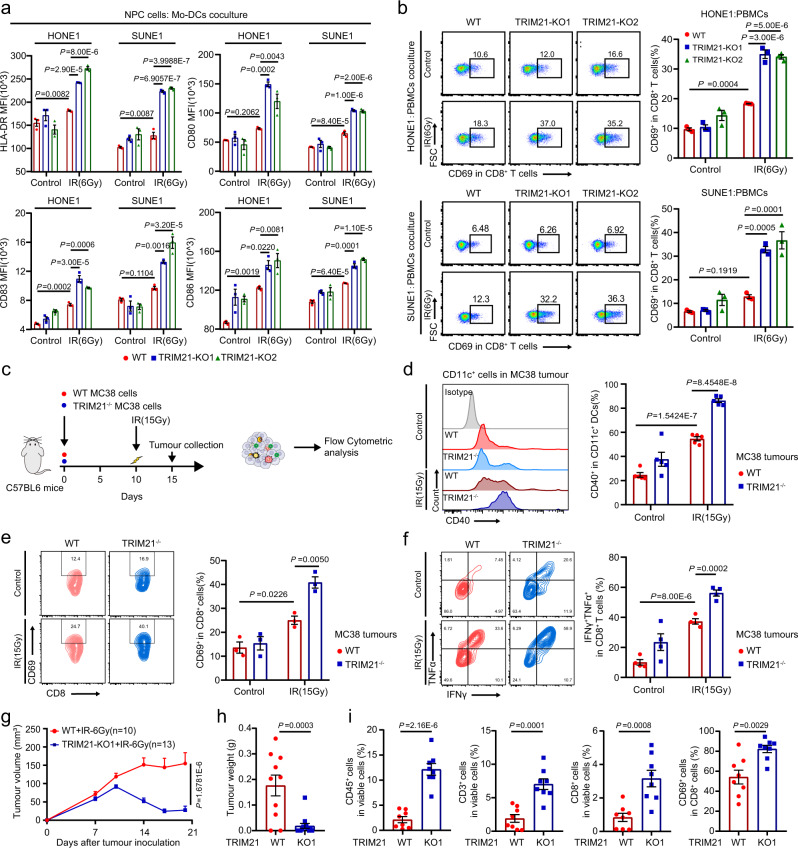
TRIM21 depletion enhances radiation-induced antitumour immunity. **a**, **b** WT and TRIM21-KO NPC cells were treated with or without IR. After 24 h, (**a**) NPC cells were cocultured with Mo-DCs for another 48 h. Then, the expression of maturation markers, including HLA-DR, CD80, CD83, and CD86, on Mo-DCs was measured by flow cytometry. **b** WT and TRIM21-KO NPC cells were cocultured with PBMCs for 24 h. The proportion of CD69^+^ cells among CD8^+^ T cells was determined by flow cytometry (the data are presented as the mean ± SEM of three independent experiments; one-way ANOVA with Tukey’s test for multiple comparisons). **c**–**e** Established WT or TRIM21^−/−^ MC38 tumours (from inoculation with 1 × 10^6^ cells) in C57BL/6 mice were subjected to focal radiation with a single fraction of 15 Gy. Tumours were harvested 5 days after IR. The surface expression level of CD40 on CD11c^+^ DCs (**d**, *n* = 5), the expression level of the activation marker CD69 on CD8^+^ T cells (**e**, *n* = 3), and the expression levels of IFN-γ and TNF-α in CD8^+^ T cells after stimulation with PMA and ionomycin (**f**, *n* = 4) were determined by flow cytometry (one-way ANOVA with the Tukey’s test for multiple comparisons). **g**–**i** WT and TRIM21-KO SUNE1 tumours were established in humanised NSG mice. Tumours were treated twice (on days 10 and 13 after tumour cell inoculation) with focal radiation (6 Gy). The tumour volume (**g**, two-way ANOVA), tumour weight (**h**, *n* = 10 and *n* = 13 in WT and TRIM21-KO1 groups respectively, two-tailed unpaired *t* test), and proportions of tumour-infiltrating CD45^+^ immune cells, CD3^+^ T cells, and CD8^+^ T cells (**i**, *n* = 8, two-tailed unpaired *t* test) are shown. The data are presented as the mean ± SEM.

As DC maturation and CD8^+^ T-cell activation are key events in antitumour immunity, we examined the maturation of peripheral monocyte-derived DCs (Mo-DCs) and the activation of CD8^+^ T cells after coculture with IR-treated NPC cells. Knockout of TRIM21 in NPC cells resulted in increased expression of maturation markers, including HLA-DR, CD80, CD83 and CD86, on the surface of Mo-DCs (Fig. 6a and Supplementary Fig. 7a). Knockout of VDAC2 in NPC cells abolished the TRIM21 deficiency-mediated upregulation of HLA-DR, CD80, CD83 and CD86 on the surface of Mo-DCs (Supplementary Fig. 7b, c). Moreover, the proportion of CD8^+^ T cells expressing the activation marker CD69 was markedly increased when CD8^+^ T cells were cocultured with IR-treated TRIM21-KO NPC cells, and this increase was reversed by VDAC2 knockout (Fig. 6b and Supplementary Fig. 7d).

We further validated the in vivo function of TRIM21 in DC maturation and CD8^+^ T-cell activation using the MC38 mouse model (Fig. 6c). Flow cytometry revealed that TRIM21 deficiency increased the proportion of CD40^+^ CD11c^+^ tumour-infiltrating DCs (Fig. 6d). In addition, the proportion of CD69^+^ CD8^+^ T cells was greatly increased in tumours with TRIM21 depletion (Fig. 6e). Moreover, CD8^+^ T cells in TRIM21-deficient tumours expressed higher levels of IFN-γ and TNF after stimulation with phorbol 12-myristate 13-acetate (PMA) and ionomycin (Fig. 6f). These findings confirm the enhancement of CD8^+^ T-cell-mediated antitumour immunity in TRIM21-deficient tumours treated with IR.

**Fig. 7:**
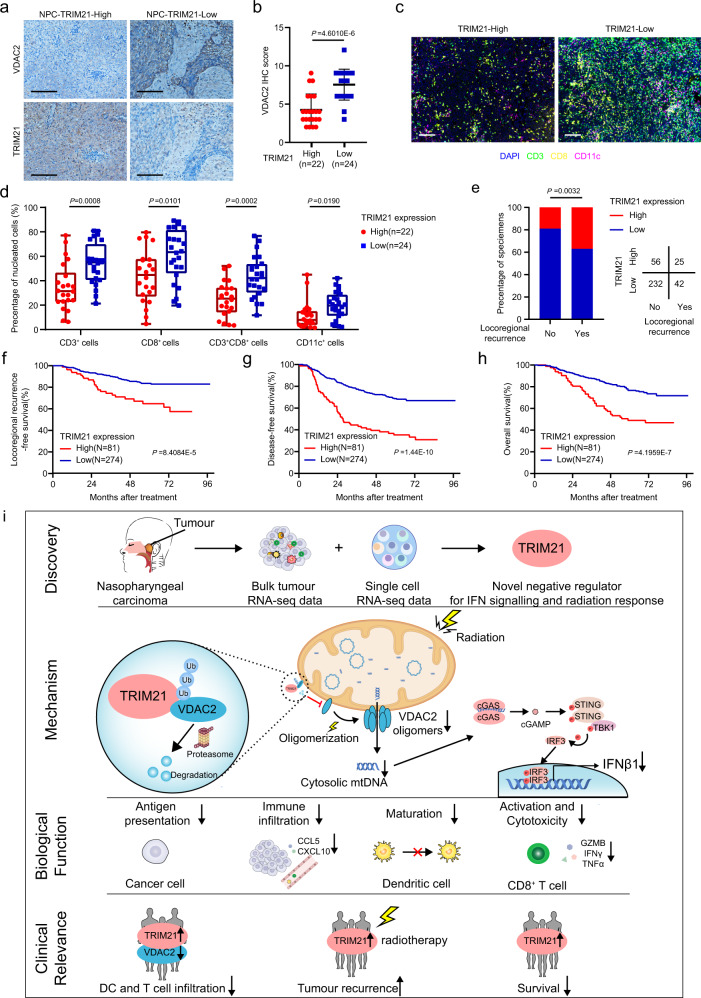
TRIM21 overexpression indicates poor survival and is associated with tumour relapse. **a** TRIM21 and VDAC2 protein expression was evaluated by IHC staining in 46 NPC tumour tissues (scale bar, 50 μm). **b** VDAC2 IHC scores in NPC tissues with high and low TRIM21 expression. The data are presented as the mean ± SD; two-tailed Mann–Whitney test). **c**, **d** Representative images (scale bar, 100 μm) and quantitative results of multiplex immunohistochemistry for CD3^+^, CD8^+^, CD3^+^CD8^+^ and CD11c^+^ cells in 46 NPC tissues with high and low TRIM21 expression (the data are presented as the mean ± SEM; two-tailed unpaired *t* test). **e** Association between TRIM21 expression and locoregional recurrence status after radical chemoradiotherapy in a cohort of 355 NPC samples (two-sided *χ*^2^ test). **f**–**h** Kaplan–Meier analysis of locoregional recurrence-free (**f**), disease-free (**g**) and overall (**h**) survival based on the TRIM21 expression level (log-rank test). **i** Proposed working model of TRIM21. Bulk and scRNA-seq revealed that TRIM21 acts as a negative regulator of IFN signalling and the radiation response in NPC cells. Mechanistically, TRIM21 facilitates the ubiquitination and degradation of VDAC2 and inhibits pore formation by VDAC2 oligomers for mtDNA release, thus suppressing radiation-induced STING–type-I IFN signalling and antitumour immune responses. High TRIM21 expression is associated with impaired antitumour immunity and poor survival in NPC patients.

To explore whether TRIM21 impairs antitumour immunity in NPC in vivo, we generated NPC xenografts with TRIM21-KO or -WT SUNE1 cells in humanised NOD/SCID/IL2rγ null mice (Supplementary Fig. 7e, f). Similarly, TRIM21 deficiency was associated with reduced tumour growth and a lower weight of the excised tumours after IR treatment (Fig. 6g, h and Supplementary Fig. 7g). The percentages of infiltrating CD45^+^ cells, CD3^+^ T cells, CD8^+^ T cells, and CD69^+^ CD8^+^ T cells were increased in irradiated TRIM21-deficient tumours (Fig. 7i). Taken together, the above findings demonstrate that TRIM21 deficiency in tumour cells could enhance antitumour immunity through the recruitment and activation of immune cells, which effectively promotes tumour radiosensitivity.

### TRIM21 overexpression indicates poor survival and is associated with tumour relapse

Finally, to explore the clinical relevance and significance of TRIM21, we first performed immunohistochemical (IHC) staining in 46 NPC tissue samples and found that the expression of TRIM21 was negatively associated with VDAC2 expression (Fig. 7a, b). Moreover, we conducted multiplex IF to detect CD3^+^, CD8^+^ and CD11c^+^ cells in these 46 NPC tissues. The results revealed that tumours with higher TRIM21 or lower VDAC2 expression levels were infiltrated with fewer CD3^+^ CD8^+^ T cells and CD11c^+^ DCs, indicating weaker antitumour immunity (Fig. 7c, d and Supplementary Fig. 8a).

We further performed IHC staining in a cohort of 355 paraffin-embedded NPC samples (Supplementary Fig. 8b and Supplementary Table 3) and found that NPC patients who developed locoregional recurrence had higher TRIM21 expression levels than those who remained tumour-free (Fig. 7e). We dichotomised the NPC patients into a high TRIM21 expression group and a low TRIM21 expression group for Kaplan–Meier analysis, which revealed that the patients with high TRIM21 expression had significantly shorter locoregional recurrence-free survival, disease-free survival, and overall survival times (Fig. 7f–h). Multivariable Cox regression analysis showed that TRIM21 expression is an independent factor for poor prognosis in NPC (Supplementary Fig. 8c–e). Collectively, our results demonstrate that high TRIM21 expression indicates a poor antitumour immune response within NPC tumours, as well as a poor survival prognosis and a high likelihood of relapse in NPC patients.

## Discussion

Radiotherapy is the primary treatment strategy for NPC because it is highly sensitive to radiation^41^. Combined chemoradiotherapy has substantially improved survival^42,43^, but about 20% of patients experience tumour relapse due to radioresistance^44,45^. Oncoproteins (i.e., MYC and β-catenin)^46,47^ and tumour suppressor proteins (i.e., USP44)^48^ have been reported to be involved in NPC radioresistance. Accumulating evidence shows that radiotherapy-induced antitumour effects are initiated by the activation of both innate and adaptive immune responses^2^. However, little is known about the mechanisms that impair radiation-induced antitumour immunity. Here, we found that tumour-intrinsic E3 ligase TRIM21 inhibited radiation-induced mtDNA release and limited antitumour immunity in NPC.

TRIM21 plays a paradoxical role in tumourigenesis and the progression of different types of cancers. TRIM21 sustains the hyperactivation of mTOR signalling by degrading ribonuclease inhibitor 1 and promotes treatment resistance^15^. In contrast, TRIM21 inhibits fatty acid synthesis to against cancer cell growth by degrading fatty acid synthase^49^. Our data revealed that TRIM21 impaired radiation-induced antitumour immunity and facilitated radioresistance in NPC and also confirmed the relationship between high TRIM21 expression and poor prognosis in NPC patients. These observations support the oncogenic role of TRIM21 in NPC progression and provide a potential biomarker for NPC treatment. TRIM21 also plays an essential role in the regulation of innate immunity as an intracellular antibody receptor that detects antibody-opsonized pathogens and contributes to the direct degradation of virions, as well as the production of proinflammatory cytokines, antigen presentation, and T-cell activation, during viral infection^30,50^. However, TRIM21 has also been reported to act as a negative regulator of type-I IFN signalling. Both proteasomal and autophagic degradation of IRF3 mediated by TRIM21 have been reported^51^, and therefore TRIM21 might have the ability to simultaneously inhibit dsDNA- and dsRNA-mediated antitumour immune response during radiation^52,53^. More recently, TRIM21 is reported to promote the degradation of intracellular dsDNA sensors, including IFI16^24^ and DDX41^21^. Our present study showed that in NPC cells, TRIM21 inhibited mtDNA sensing and type-I IFN responses by suppressing mtDNA release during radiotherapy instead of by directly regulating the STING pathway, which broadens our knowledge of the role of TRIM21 in STING–type-I IFN signalling-mediated antitumour immunity. Due to the dual role of TRIM21 in impairing dsDNA sensing in myeloid DCs and mtDNA sensing in tumour cells, pharmacological interventions targeting TRIM21 may simultaneously activate mtDNA sensing and IFN responses in both tumour cells and DCs, leading to further enhancement of the antitumour immune response.

Recent studies highlight the role of mtDNA in radiation-induced antitumour immunity. In breast cancer and colon adenocarcinoma, radiation-induced mtDNA accumulation in the cytoplasm was found to induce the activation of STING signalling and subsequent type-I IFN production in tumour cells, which triggered a potent antitumour immune response^26,27,54^. Correspondingly, our findings demonstrated that cytosolic mtDNA induced tumour-intrinsic STING signalling and type-I IFN responses, thus leading to a robust radiation-induced antitumour immune response in NPC. Moreover, we reported for the first time that VDAC2 on mitochondria plays a key role in and is directly responsible for radiation-induced mtDNA release. Mitochondrial outer membrane permeabilization is a prerequisite for mtDNA release^40^. Previous studies have reported that the mtDNA release was dependent on the BAX protein in colorectal cancer cells only when irradiated with a relatively high dose of 20 Gy but not lower doses^26^. In our study, we found that the inhibition of BAX did not impair mtDNA-mediated type-I IFN responses in irradiated NPC cells, which was attributed to that only a small fraction of cells developed apoptosis when treated with 6 Gy^48^, and thus BAX that facilitates mtDNA release in the apoptotic cells^28^ might not be fully activated and oligomerized. In addition, VDAC1/3 oligomers have been reported to facilitate mtDNA release under mROS stimulation^40^. However, we found that the inhibition of VDAC1 did not impair mtDNA-mediated type-I IFN responses. In contrast, we observed that VDAC2, a previously reported BAX/BAK interactor^29^, formed dimers and higher-order oligomers after radiation exposure. This observation is consistent with previous findings indicating that both VDAC1 and VDAC2 can oligomerize under increasing levels of mROS^28,39^, which are usually generated by radiation for tumour killing^55^. Moreover, elevated mROS has been revealed to break large intact mtDNA molecules into short and unbound fragments that can pass through the mitochondrial pores formed by VDAC oligomers^40^. Thus, inhibition of VDAC2 oligomerization by DIDS reduced cytosolic mtDNA accumulation and sensing. Our data strongly suggest that VDAC2 oligomers facilitate mtDNA release in irradiated NPC cells.

VDAC2 is well-documented to regulate different modes of cell death, including apoptosis and ferroptosis. VDAC2 was first reported to be able to interact with the inactive conformer of BAK, resulting in the inhibition of BAK oligomerization-mediated mitochondrial apoptosis^34^. Subsequent studies elucidated that VDAC2, by helping BAX to translocate to and oligomerize on the mitochondrial membrane to mediate apoptosis, acts as a tumour suppressor^35,56^. In addition, VDAC2 is a target of erastin, a ferroptosis activator^57^, and degradation of VDAC2 promotes tumour resistance to erastin-induced ferroptosis^58^. In the current study, we revealed the novel role of VDAC2 in regulating mtDNA release via oligomerization in irradiated NPC cells. By facilitating mtDNA release, VDAC2 facilitated radiation-induced antitumour immunity, and a positive correlation was established between VDAC2 expression and CD8^+^ T-cell antitumour immunity. A previous study reported that VDAC2 can be ubiquitinated by Nedd4, but this ubiquitination occurred only under erastin treatment^58^. In our investigation, ubiquitination of VDAC2 by TRIM21 seemed to be a general phenomenon and was independent of radiation exposure. This finding suggests that VDAC2-related biological processes might be widely influenced by TRIM21. Thus, targeting TRIM21 might be a practical strategy to activate VDAC2-mediated apoptosis and antitumour immune responses.

In summary, our study reveals that the tumour cell-intrinsic E3 ligase TRIM21 inhibits radiation-initiated, VDAC2 dependent, and mtDNA-induced STING–type-I IFN signalling and antitumour immune responses in NPC (Fig. 7i). Given that depletion of TRIM21 can potentiate radiation-induced antitumour immunity, either genetic deletion or pharmacological inhibition of TRIM21 may be a promising therapeutic strategy for cancer.

## Methods

### Clinical specimens

We collected 46 fresh-frozen NPC samples for TRIM21 and VDAC2 protein expression as well as the tumour immune infiltration analysis, and collected 355 paraffin-embedded NPC samples for survival analysis from the Sun Yat-sen University Cancer Center (Guangzhou, China) between January 2006 and December 2010. None of the patients had received any antitumour therapy before sampling. Tumour pathological types were classified according to the WHO classification, and tumour–node–metastasis (TNM) stages were reclassified based on the 7th edition of the American Joint Committee on Cancer (AJCC) Cancer Staging Manual. All patients received radical radiotherapy and platinum-based chemotherapy. The patients’ clinical characteristics are listed in Supplementary Table 3. The Institutional Ethical Review Boards of Sun Yat-sen University Cancer Center approved this study (B2022-259-01), in which anonymized data were analysed, and waived the requirement for informed consent.

### Cell culture and treatment

The human NPC cell lines HONE1 and SUNE1 were provided by Professor Mu-Sheng Zeng at Sun Yat-sen University Cancer Center (Guangzhou). MC38 murine colon adenocarcinoma cells and HEK293T cells were obtained from the American Type Culture Collection (ATCC). All cell lines were cultured in RPMI-1640 medium or DMEM (Invitrogen) supplemented with 10% foetal bovine serum (FBS; Gibco) and 100 U/ml penicillin–streptomycin (Gibco, Cat# 15140122). We knocked out the TRIM21, VDAC2, or VDAC1 genes by CRISPR/Cas9 genome editing with PX458 plasmid^40^. The sequences of the small guide RNAs (sgRNAs) are listed in Supplementary Table 4.

Cells were seeded in a culture plate one day prior to treatment with radiation or an inhibitor after complete adherence. NPC and MC38 cells were, respectively, treated with a single fraction of 6 or 15 Gy IR, unless otherwise indicated, using the RS-2000-PRO-225 Biological Irradiator (1.827 Gy/min). Cells were treated with the proteasome inhibitor MG132 (10 μM, Sigma) or the autophagy inhibitor CQ (50 μM, Sigma-Aldrich) for 6 h or with the protein synthesis inhibitor CHX (100 μg/ml, Sigma) for 0, 2, 4, 8 or 12 h. For the inhibition of BAX or VDAC2 oligomerization, cells were treated with BAI1 (2 μM, Selleck) or DIDS (100 μM, TargetMol) for 24 h before IR treatment.

### Plasmids and transfection

The TRIM21 or VDAC2 coding sequence was cloned into the pSin-EF2-puro and pCMV-CFP/YFP vector to construct the following plasmids: pSin-EF2-puro-TRIM21-FLAG, pSin-EF2-puro-TRIM21-MYC, pSin-EF2-puro-VDAC2-FLAG, pSin-EF2-puro-VDAC2-HA, pSin-EF2-puro-VDAC2-MYC, pCMV-TRIM21-CFP, and pCMV-VDAC2-YFP. PRK-HA-Ub (K48 or K63) was a gift from Professor Bo Zhong (Wuhan University). The shRNA sequences targeting STING and VDAC3 were obtained via the shRNA sequence prediction website Portals (Supplementary Table 4) and were then synthesised and inserted into the pLKO.1-RFP vector to construct the PLKO.1-shSTING or PLKO.1-shVDAC3 plasmid. Transfections were performed using Lipofectamine 3000 (Invitrogen) according to the manufacturer’s instructions, and the transfection efficiency was determined by RT–qPCR and western blotting after 24–48 h of transfection.

### RT–qPCR

Total RNA was extracted using TRIzol reagent (Invitrogen). Complementary DNA was synthesised using HiScript III RT SuperMix for qPCR (+gDNA wiper) (Vazyme, R323-01), and qPCR was performed using ChamQ SYBR qPCR Master Mix (Vazyme, Q311-03) on a CFX96 Touch sequence detection system (Bio-Rad) or a LightCycler 480 System (Roche). Relative gene expression was calculated by the 2^-ΔΔCT^ method with *GAPDH* as the internal control. The primer sequences are listed in Supplementary Table 4.

### ELISA

The IFNβ1 concentration was quantified with an IFN-β ELISA kit (DIFNB0, R&D Systems) according to the manufacturer’s protocol. Absorbance was read using an 800 TS microplate reader (BioTek). IFNβ1 concentrations between the limit of detection and limit of quantification were considered reliable only when the standard curves remained linear (*r*^2^ ≥ 0.99). IFNβ1 concentrations that were undetectable or below the detection limit were recorded as 0 pg/ml.

### Western blot analysis

Cells were lysed with 1× RIPA lysis buffer (Millipore) supplemented with protease and phosphatase inhibitors (Roche), and the lysates were then sonicated to obtain total protein. Total protein was separated by SDS–PAGE and transferred to PVDF membranes (Millipore). The membranes were blocked with 5% bovine serum albumin and incubated first with primary antibodies and then with HRP-conjugated secondary antibodies. Greyscale analysis of immunoblot bands was performed using ImageJ software. The antibodies used are listed in Supplementary Table 5, and the unprocessed scans of the immunoblots are provided in the Source data.

### Clonogenic assay

In all, 200–1000 WT and TRIM21^−/−^ MC38 cells were seed onto the six-well plates and treated with IR (0, 5, 10, 15 and 20 Gy). Cells were grown for 12 days to develop single-cell colonies, and then, were fixed with methanol and stained with crystal violet. Colonies containing more than 50 cells were counted. Cell survival curves were fitted according to the equation: surviving fraction (*SF*) = *exp (−αD* − *βD2)*.

### Flow cytometric analysis

Harvested cells were washed twice with ice-cold PBS. Fixable Viability Dye eFluor™ 455UV or eFluor® 780 (eBioscience; 65-0868-14 and 65-0865-14) was used to label dead cells, and an Intracellular Fixation & Permeabilization Buffer Set (eBioscience, 88-8824-00) was used for intracellular staining according to the manufacturer’s instructions. Cells were incubated with an FcR-blocking reagent (Biolegend) to avoid nonspecific binding, suspended in flow staining buffer, and incubated with the indicated antibodies. After staining, the cells were washed, centrifuged and suspended in flow staining buffer. All data were obtained with a CYTOFLEX flow cytometer (Beckman Coulter), and the results were analysed using Flow Jo software (Tree Star). The antibodies used are listed in Supplementary Table 5, and the flow cytometry gating strategy is provided in Supplementary Fig. 9.

### IF and IHC analyses

Cells were fixed with 4% paraformaldehyde and permeabilized with 0.1% Tween 20 and 0.5% Triton X-100. For dsDNA staining, cells were permeabilized with 0.01% Triton X-100^25^. For mitochondrial staining, cells were prestained with MitoTracker Red CMXRos (Beyotime, C1035). Then, the cells were blocked with QuickBlock™ Blocking Buffer for Immunol Staining (Beyotime, P0260) and incubated with primary antibodies overnight at 4 °C. The cells were then stained with Hoechst, and images were acquired with an LSM980 confocal laser scanning microscope operated with ZEN (blue edition) software (Zeiss). dsDNA and TFAM were quantitatively analysed from 30 cells in ≥15 randomly selected fields of view. Multiplex IF was performed using a PANO 7-plex IHC Kit (Panovue) according to the manufacturer’s instructions. Tumour-infiltrating CD3^+^ T cells, CD8^+^ T cells, CD3^+^CD8^+^ T cells, and CD11c^+^ DCs were analysed using the HALO image analysis platform. IHC staining was analysed, and staining intensity values were calculated as reported in our previous study^48^. The antibodies used are listed in Supplementary Table 5.

### mtDNA depletion

Cells were cultured in DMEM supplemented with 500 ng/ml ethidium bromide (E1510, Sigma-Aldrich), 1 mM sodium pyruvate (Gibco, 11360070), and 50 μg/ml uridine for 10 days to obtain mtDNA-depleted (rho^0^) cells^26^. Total gDNA and mtDNA were extracted with a TIANamp Micro DNA Kit (TIANGEN Biotech). The efficacy of mtDNA depletion was verified by the decrease in the abundance of *MT-ATP8* DNA sequences.

### Quantification of mtDNA release

Cells were resuspended in 170 μl of digitonin buffer containing 150 mM NaCl (Bio Sharp, BL542A), 50 mM HEPES (Beyotime, C0217), and 25 μg/ml digitonin (Beyotime, ST1272), as previously reported^40^. The homogenates were incubated and centrifuged. A 1:15 dilution of the supernatant (cytosolic mtDNA) was used for qPCR. The pellet was used for the extraction of total DNA with the TIANamp Micro DNA Kit (TIANGEN Biotech). The amount of cytosolic mtDNA in the supernatant was normalised to the amount of total mtDNA in the pellet.

### mPTP measurement

The mPTP opening was measured using the mPTP Assay Kit (Beyotime) according to the manufacturer’s instructions. Briefly, NPC cells treated as indicated were digested, washed with PBS, and loaded with calcein AM and CoCl2 for 30 min at 37 °C. After washing twice, fluorescence intensity was determined by flow cytometry.

### Co-IP and MS analysis

Cells were lysed on ice with IP lysis buffer supplemented with protease and phosphatase inhibitors. The lysates were immunoprecipitated with the indicated antibodies (3 μg) overnight at 4 °C. Pierce^TM^ Protein A/G Magnetic Beads (Thermo Scientific) were used to capture the immune complexes, which were washed with IP wash buffer. The eluates were then separated by SDS–PAGE and stained with a Fast Silver Stain Kit (Beyotime, P0017S). MS was conducted by Wininnovate Biotechnology. The proteins of interest in the co-IP products were detected by Western blotting. The ubiquitin assay was conducted under denaturing conditions as previously described^48^. The antibodies used are listed in Supplementary Table 5.

### FRET assay

TRIM21-CFP and VDAC2-YFP expressing plasmids were constructed and transfected into HEK293T cells. After 24 h, cells were treated with MG132 for 6 h to prevent the degradation of VDAC2. After being washed and fixed, cells were subjected to FRET assay in a Zeiss LSM980 confocal microscope. FRET was quantified by acceptor (YFP) photobleaching^59^. Acceptor (YFP) photobleaching was progressively performed in regions of the 20 cells where TRIM21-CFP and VDAC2-YFP could be observed to colocalize. Photobleaching was automated with Zeiss software and manually stopped when the intensity of donor (CFP) was no longer increased. Images were acquired after every photobleaching. The FRET efficiency (E), which was calculated from the donor (CFP) initial (F_prebleach_) and final (F_postbleach_) fluorescence values, according to the equation E = 1 − (F_prebleach_/F_postbleach_).

### Crosslinking assay

Cells were collected, washed, and suspended in 200 μl of PBS containing 5 mM DSS (Thermo Scientific). The reaction mixture was incubated at 37 °C for 30 min and quenched with 20 mM Tris-HCl for 15 min. Cells were harvested, lysed and analysed by western blotting.

### DC maturation and CD8^+^ T-cell activation assays

Peripheral blood monocytes (PBMCs) were isolated using CD14 magnetic microbeads (Miltenyi Biotec, 130-050-201). Mo-DCs were generated by culturing monocytes in RPMI-1640 medium supplemented with 25 ng/ml GM-CSF (R&D, 215-GM-010), 10 ng/ml IL-4 (204-IL-010), 10% FBS, and 100 U/ml penicillin–streptomycin for 6 days. NPC cells were then treated with IR as indicated. After 24 h, NPC cells were cocultured with Mo-DCs or PBMCs for the indicated time. Mo-DC maturation was confirmed by the expression of CD11c, HLA-DR, CD80, CD83 and CD86 expression as determined by flow cytometry. CD8^+^ T-cell activation was confirmed by flow cytometric detection of CD8^+^CD69^+^ T cells.

### Tumour growth, treatment and analyses

Animal experiments in this study were approved by the Experimental Animal Ethics Committee, Sun Yat-sen University Cancer Center (L025501202108037). Animals were co-housed and all experiments were performed in the Animal Experiment Center of Sun Yat-sen University Cancer Center. Six-week-old female, specific pathogen-free/SPF, BALB/c nude mice (A196) and C57BL/6 mice (A416) were purchased from Charles River Laboratories (Zhejiang). BALB/c nude mice were subcutaneously inoculated with 1 × 10^6^ WT or *TRIM21*-KO SUNE1 cells. C57BL/6 mice were subcutaneously inoculated with 5 × 10^5^ or 1 × 10^6^ WT or *TRIM21*-KO (*TRIM21*^*−/−*^) MC38 cells. After random grouping of mice, the mice were anaesthetised with isoflurane and fixed, the mice body were covered with lead plates (>5 mm), and then the tumours were locally irradiated with a single fraction (6 Gy for SUNE1 tumours and 15 Gy for MC38 tumours) using an RS-200-PRO-225 Biological Irradiator (1.827 Gy/min) on day 10 after cell inoculation. The anti-CD8α antibody (100 μg per mouse, BioXcell, BE0004) or anti-IFNAR1 antibody (100 μg per mouse, BioXcell, BE0241) was administered intraperitoneally three times on days 7, 14 and 21. The tumours were monitored every three days. Mice were euthanized by CO_2_ asphyxiation at the indicated time points for tumour harvesting or after the appearance of tumours with a diameter greater than 1.5 cm in any group. The weights of the excised tumours were recorded. The tumour tissues were digested into single-cell suspensions, and infiltrating immune cells were analysed by flow cytometry. To evaluate the function of CD8^+^ T cells, single-cell suspensions from MC38 tumours were treated with Cell Stimulation Cocktail (plus protein transport inhibitors) (00-4975-93, Thermo Scientific) for 4 h before staining, and the percentages of IFNγ^+^TNFα^+^ CD8^+^ T cells were determined. To evaluate the abscopal effects, 5 × 10^5^ WT or *TRIM21*^*-/-*^ MC38 cells were subcutaneously inoculated into the right flanks of C57BL/6 mice on day 0 to obtain the primary tumours, and 3 × 10^5^ WT-MC38 cells were inoculated into the left flank on day 2 to generate the abscopal tumours, respectively. The primary tumours were subjected to local radiation at a single fraction of 15 Gy on day 10, while the abscopal tumours on the left flank were shielded from radiation. The primary and abscopal tumours were monitored and recorded every 3 days.

A 6-week-old female, SPF humanised NSG mouse model (Shanghai Model Organisms, DS008) was established by tail vein injection of human PBMCs (5 × 10^6^) and validated by the detection of more than 1% human CD45^+^ cells in the peripheral blood of the mice 1 week after injection. The mice were then subcutaneously inoculated with 1 × 10^6^ WT or *TRIM21*-KO SUNE1 cells. Tumours were irradiated twice (on days 10 and 13) and monitored every three days. One week after the last radiation, the tumours were harvested for weighing and flow cytometric analysis.

### Bioinformatics analysis

We determined the CD8^+^ T-cell antitumour immunity of 128 NPC tumours, based on their bulk tumour RNA-seq data^18^, by generating a score that reflects the CD8^+^ T-cell infiltration and antitumour immune response for each sample. The score was calculated through the single sample Gene Set Enrichment Analysis on GenePattern (https://cloud.genepattern.org/) and the CD8^+^ T-cell-related genes published in ref. ^19^ were used as the gene sets. Differentially expressed genes between NPC tumours with high (top 10%, *n* = 13) and low (bottom 10%, *n* = 13) CD8^+^ T scores were identified (log2FC ≥ 1.5, FDR < 0.01). CIBERSORTx and GSEA (version 4.0.2) were used to estimate tumour immune infiltration and identify differences in gene signatures between tumours with high (top 10%, *n* = 13) and low (bottom 10%, *n* = 13) TRIM21 expression. We then selected 1432 NPC tumour cells with detectable TRIM21 expression from our previous scRNA-seq data^18^. Genes that were highly expressed (FC ≥ 2, *P* < 0.01) in TRIM21-high NPC cells (top 10% vs. bottom 10%, *n* = 143 cells per group) were used for GO term enrichment analysis (https://david.ncifcrf.gov/). The results were visualised with R Studio or a free online platform for data analysis and visualisation (http://www.bioinformatics.com.cn).

### Statistical analysis and reproducibility

Data are presented as the mean ± SEM of at least three independent experiments. The boxplots indicate median (center), 25th and 75th percentiles (bounds of box), and minimum and maximum (whiskers). Statistical analyses were performed using either GraphPad Prism 8 (GraphPad) or SPSS Statistics version 25 (IBM). Two-tailed unpaired Student’s *t* test and one-way or two-way ANOVA with the Tukey’s test for multiple comparisons were used to calculate *P* values. Time-to-event data were described using Kaplan–Meier curves, and differences in survival were determined using the log-rank test. The chi-square (*χ*^2^) test or a nonparametric test was used to compare clinical characteristics. A multivariable Cox proportional hazards model was used to estimate independent prognostic factors. A *P* value of <0.05 was considered statistically significant.

### Reporting summary

Further information on research design is available in the Nature Portfolio Reporting Summary linked to this article.

## Supplementary information

Supplementary information

Reporting Summary

## Data Availability

The NPC sequencing data used in this study was previously published (https://www.ncbi.nlm.nih.gov/geo/query/acc.cgi?acc=GSE150430)^18^. All the other data supporting the findings of this study are available within the article and its Supplementary Information files. All original data for this study can be obtained from the corresponding author. The authenticity of this article has been validated by uploading the key raw data onto the Research Data Deposit public platform (www.researchdata.org.cn), with the approval RDD number as RDDB2023358879. Source data are provided with this paper.

## Source data

Source data
