# Supplementary material for: TRIM21 inhibits irradiation-induced mitochondrial DNA release and impairs antitumour immunity in nasopharyngeal carcinoma tumour models
Source: Nat Commun. 2023 Feb 16;14:865. doi: 10.1038/s41467-023-36523-y (PMC9935546; doi:10.1038/s41467-023-36523-y)
Supplement: Supplementary file 1 — Supplementary information [file 41467_2023_36523_MOESM1_ESM.pdf]

Supplementary Information

TRIM21 inhibits irradiation-induced mitochondrial DNA release and impairs antitumour immunity in nasopharyngeal carcinoma tumour models

Li et al.

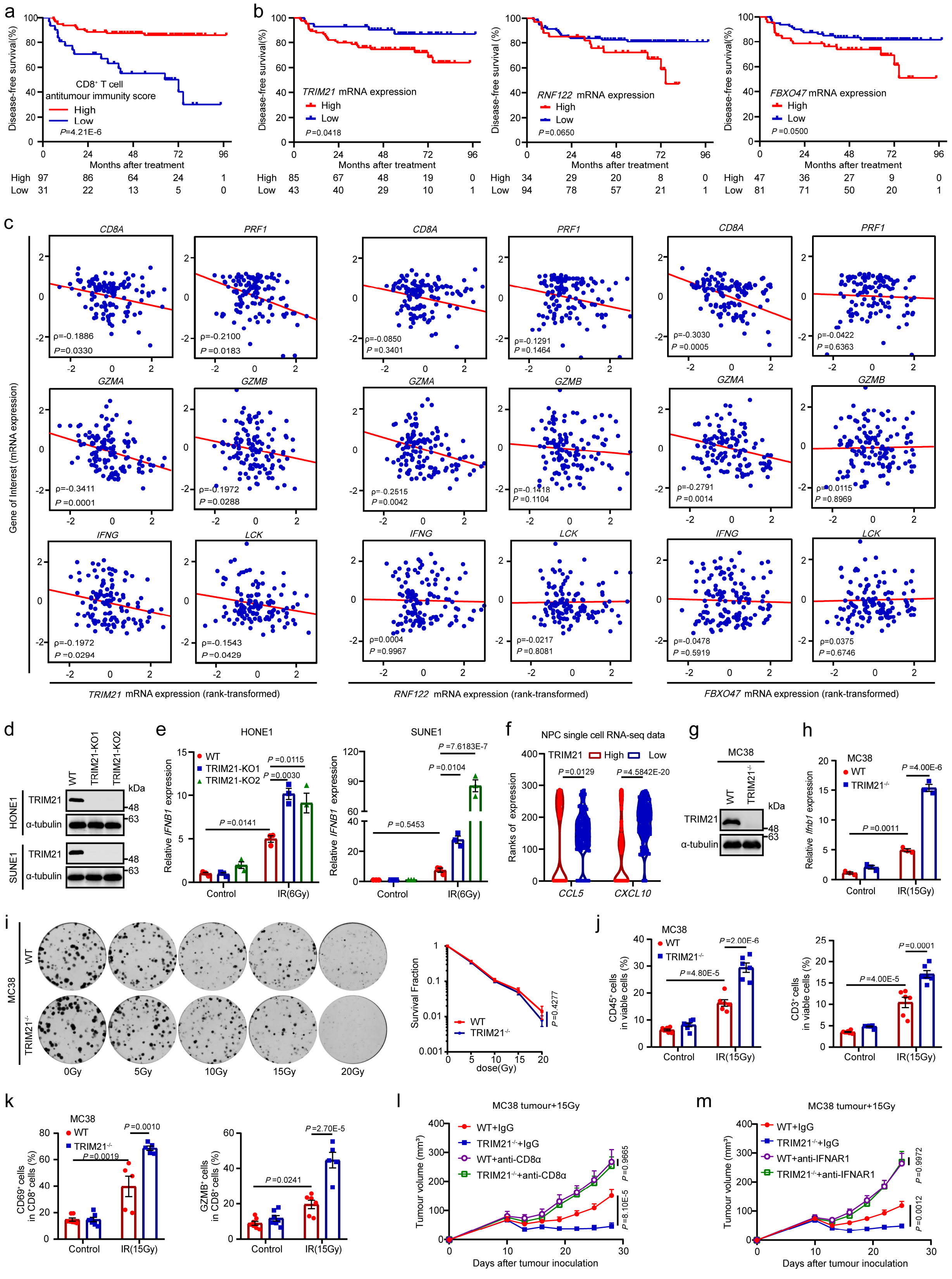

Supplementary Fig. 1. (Extended data related to Main Fig. 1)

a, Kaplan–Meier survival curves for NPC patients with high or low $CD8^+$ T-cell antitumor immunity scores (log-rank test). **b,** Kaplan–Meier survival curves for NPC patients with high or low *TRIM21*, *RNF122*, and *FBXO47* mRNA expression (log-rank test). **c,** Spearman correlation analysis (Two-tailed) of *TRIM21*,

RNF122, and *FBXO47* expression with *CD8A*, *PRF1*, *GZMA*, *GZMB*, *IFNG*, and *LCK* expression (n=128)[21]. **d,** TRIM21 expression in TRIM21-KO HONE1 and SUNE1 cells. **e,** Relative *IFNB1* mRNA expression in WT and TRIM21-KO HONE1 and SUNE1 cells 48 h after IR treatment. **f,** Ranks of *CCL5* and *CXCL10* expression in *TRIM21*-high and *TRIM21*-low NPC cells in the scRNA-seq data (n = 143 in each group, Two-tailed Mann-Whitney test). **g,** TRIM21 expression in TRIM21$^{-/-}$ MC38 cells. **h,** Relative *Ifnb1* mRNA expression in WT and TRIM21$^{-/-}$ MC38 cells 48 h after IR treatment. **i,** Clonogenic assays and survival fraction curves of WT and TRIM21$^{-/-}$ MC38 cells after exposure to the indicated IR doses (two-way ANOVA). **j-k,** Percentages of CD45^{+}, CD3^{+}, CD69^{+}CD8^{+}, and GZMB^{+}CD8^{+} cells in control and irradiated WT and TRIM2*1*$^{-/-}$ MC38 tumours (**j**, n = 7 in each control group and n = 6 in each IR group; **k**, n = 7 in each control group and n = 5 or 6 in IR- control or TRIM21-KO group respectively). **l-m**, WT and TRIM21$^{-/-}$ MC38 tumours were established in C57BL/6 mice and were subjected to focal radiation with a single fraction of 15 Gy on day 10 after tumour cell inoculation. The anti-CD8α or anti-IFNAR1 antibody (100 µg per mice) was administered intraperitoneally for three times on days 7, 14 and 21. Tumour growth was monitored (n = 6 in each group, two-way ANOVA with the Dunnett's multiple comparisons test). The results are representatives of three independent experiments (**d**, **e**, **g-i**). Data in **e**, **h-m** are shown as the mean ± SEM. Statistical analysis was performed by one-way ANOVA with the Tukey's test for multiple comparisons in **e**, **h**, **j**, and **k**.

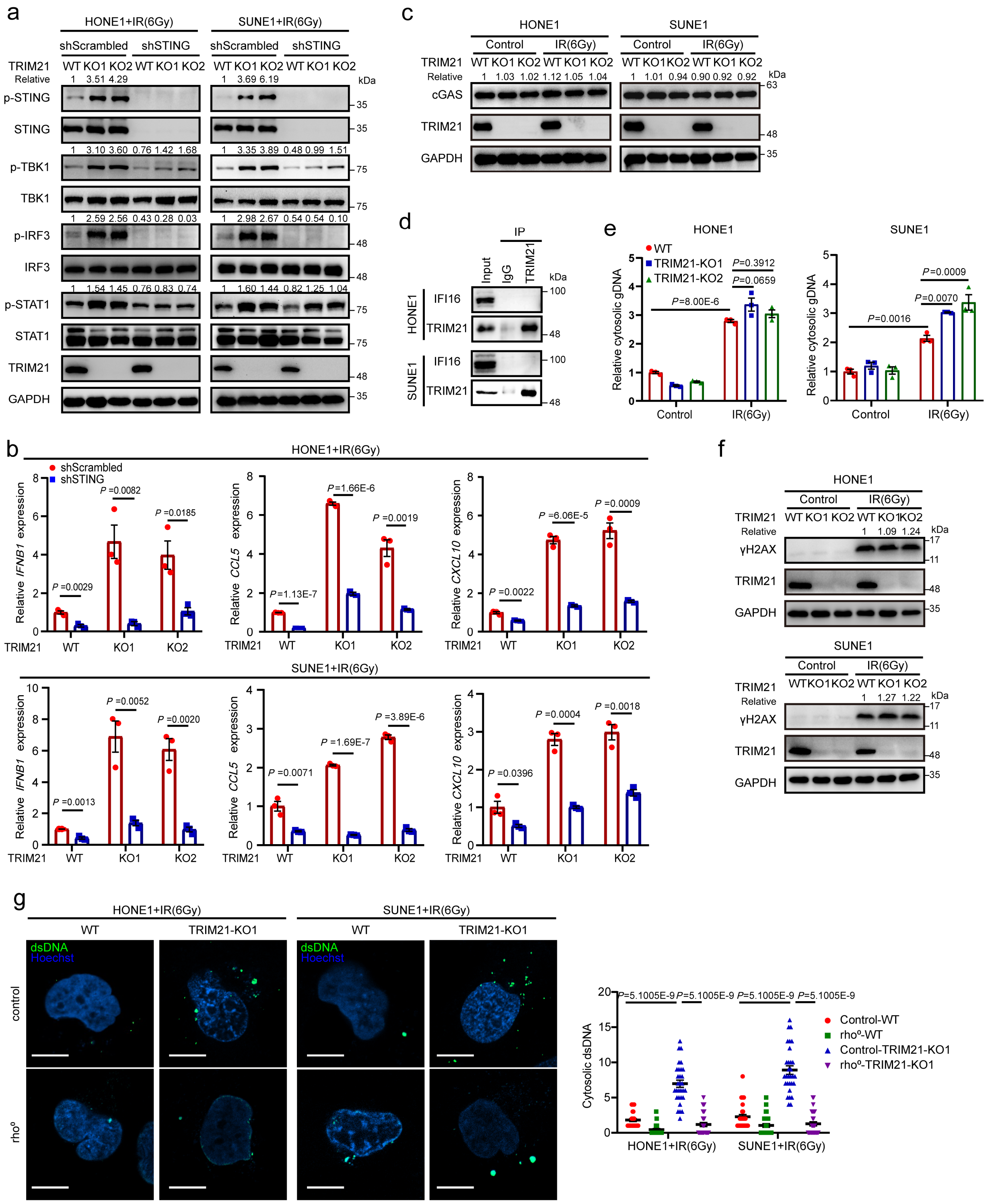

Supplementary Fig. 2. (Extended data related to Main Fig. 2)

a-b, WT and TRIM21-KO HONE1 and SUNE1 cells with stable STING knockdown were treated with IR. 48 hours later, the phosphorylation of STING, TBK1, IRF3, and STAT1 was evaluated by Western blot analysis (**a**), and the relative mRNA expression of *IFNB1*, *CCL5*, and *CXCL10* was determined by qPCR (**b**). **c,** Protein level of cGAS in WT and TRIM21-KO HONE1 and SUNE1 cells with or without IR treatment. **d,** Co-IP with an anti-TRIM21 antibody in NPC cells revealed no interaction of endogenous TRIM21 and IFI16. **e-f**, WT and TRIM21-KO NPC cells were treated with or without IR. After 24 h, The abundance of *GAPDH* DNA sequence in the cytosolic fraction was measured by qPCR (**e**), the levels of γH2AX were measured by

Western blot analysis (**f**). **g**, Cytosolic DNA accumulation in control and rho^0 cells was assessed by IF staining with a dsDNA-specific antibody at 24 hours after IR. Representative images (scale bar, 10 μm) and quantitative results are shown (n=30 cells per group). The results are representatives of three independent experiments (**a-g**) and are shown as the mean±SEM in **b**, **e** and **g**. Statistical analysis was performed by a two-tailed unpaired t test in **b**, and by one-way ANOVA with the Tukey's test for multiple comparisons in **e** and **g**.

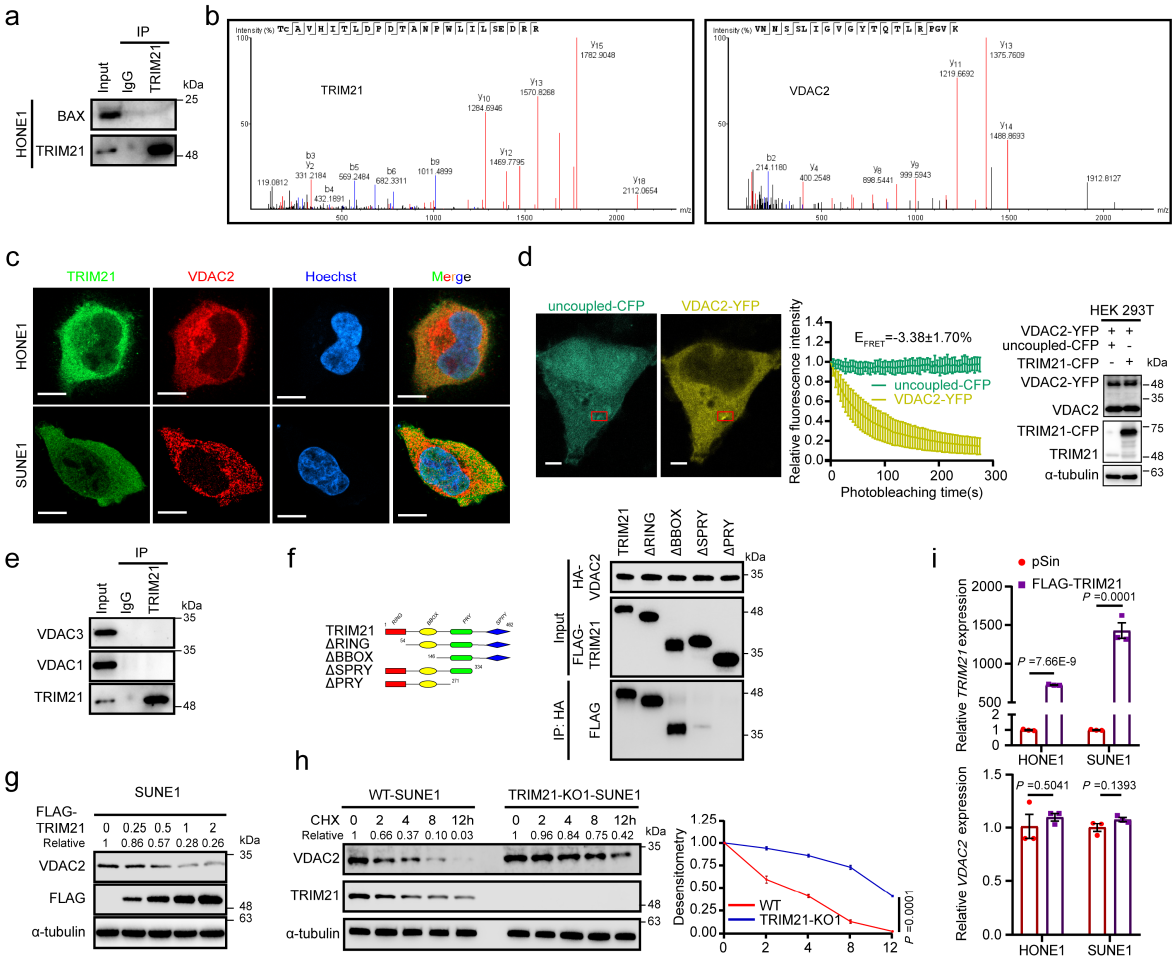

Supplementary Fig. 3. (Extended data related to Main Fig. 3)

a, Co-IP with anti-IgG or anti-TRIM21 antibody revealed no interaction of endogenous BAX and TRIM21 in HONE1 cells. **b,** TRIM21 junction-specific peptides of VDAC2 were identified by immunoprecipitation with an anti-FLAG antibody in HONE1 cells overexpressing FLAG-TRIM21. **c**, IF staining revealed the colocalization of exogenous FLAG-tagged TRIM21 (green) and HA-tagged VDAC2 (red). (Scale bars, 10 μm). **d**, Left: FRET assay in HEK293T cells transfected with uncoupled-CFP and VDAC2-YFP. Representative cell with uncoupled-CFP and VDAC-YFP, and region of FRET analysis was indicated. Scale bar: 5 μm. Middle: The exponential decrease in VDAC2-YFP fluorescence and the stable uncoupled-CFP fluorescence during YFP-photobleaching in 20 cells. The FRET efficacy (E_{FRET}) was reported. Right: The VDAC2-YFP and TRIM21-CFP or uncoupled-CFP plasmids were co-transfected into HEK293T cells for 24h. The level of indicated proteins were measured by immunoblot after 6 h treatment of MG132. **e**, Co-IP with anti-IgG or anti-TRIM21 antibody revealed the interaction of endogenous VDAC1, VDAC3 and TRIM21 in HONE1 cells. **f**, Interactions between HA-tagged VDAC2 and FLAG-tagged full-length TRIM21 and several TRIM21 truncations in HEK293T cells. **g,** VDAC2 protein level in SUNE1 cells transfected with gradient concentrations of FLAG-tagged TRIM21. **h,** Immunoblot band (left) and corresponding greyscale analysis (right) of VDAC2 in WT and TRIM21-KO SUNE1 cells treated with cycloheximide (CHX) for the indicated time. **i,** Relative *TRIM21* and *VDAC2* mRNA expression in TRIM21-overexpressing HONE1 and SUNE1 cells. The results are representatives of three independent experiments (**a**, **c-i**) and are shown as the mean± SEM (**h** and **i**). Statistical analysis was performed by two-way ANOVA in **h** and by two-tailed unpaired t test in **i**.

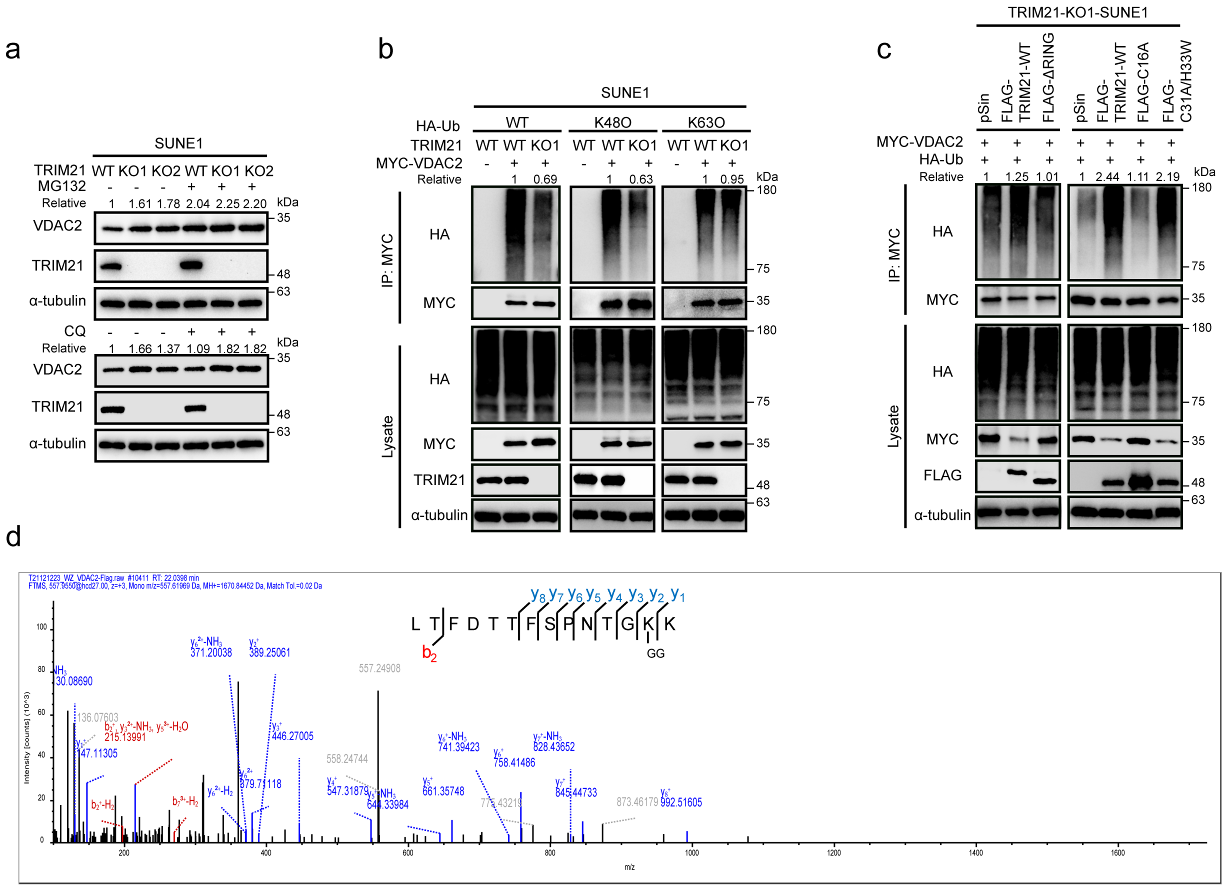

Supplementary Fig. 4. (Extended data related to Main Fig. 3)

a, VDAC2 protein level in WT and TRIM21-KO SUNE1 cells after 6 h of treatment with MG132 (top) or CQ (bottom). **b,** Ubiquitination level of MYC-tagged VDAC2 in WT and TRIM21-KO SUNE1 cells transfected with HA-Ub as indicated. **c,** Ubiquitination of MYC-tagged VDAC2 by WT TRIM21 and three TRIM21 mutants in SUNE1 cells. The results are representatives of three independent experiments (**a-c**). **d,** Mass spectrometry analysis revealed the ubiquitination site in FLAG-VDAC2.

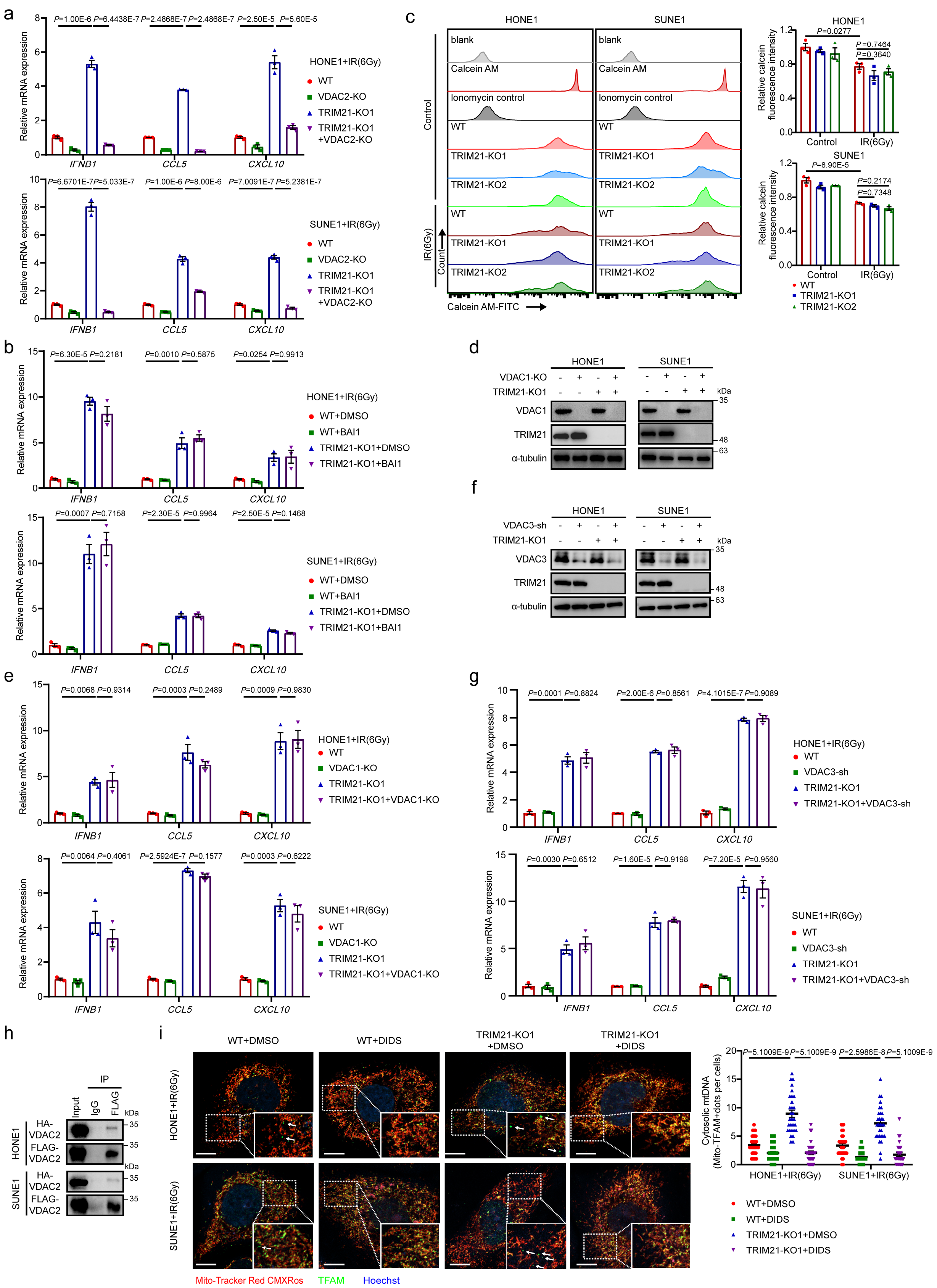

Supplementary Fig. 5. (Extended data related to Main Fig. 4)

a, Relative *IFNB1*, *CCL5*, and *CXCL10* mRNA expression in WT, VDAC2-KO, TRIM21-KO, and TRIM21-KO+ VDAC2-KO HONE1 and SUNE1 cells 48 h after IR treatment. **b,** Relative *IFNB1*, *CCL5* and *CXCL10*

mRNA expression in BAI1 (BAX inhibitor)-pretreated WT and TRIM21-KO HONE1 and SUNE1 cells 48 h after IR treatment. **c**, WT and TRIM21-KO NPC cells were treated with or without IR. After 24 h, the levels of mitochondrial permeability transition pore (mPTP) were measured by the calcein-quenching assay. Representative histogram and relative median fluorescence intensity were reported. **d,** VDAC1 expression in WT, VDAC1-KO, TRIM21-KO, and TRIM21-KO+ VDAC1-KO HONE1 and SUNE1 cells. **e,** Relative *IFNB1*, *CCL5* and *CXCL10* mRNA expression in WT, VDAC1-KO, TRIM21-KO, and TRIM21-KO+ VDAC1-KO HONE1 and SUNE1 cells 48 h after IR treatment. **f,** VDAC3 expression in WT, VDAC3-sh, TRIM21-KO, and TRIM21-KO+ VDAC3-sh HONE1 and SUNE1 cells. **g,** Relative *IFNB1*, *CCL5* and *CXCL10* mRNA expression in WT, VDAC3-sh, TRIM21-KO, and TRIM21-KO+ VDAC3-sh HONE1 and SUNE1 cells 48 h after IR treatment. **h,** Co-IP analysis revealed the interaction of FLAG-tagged and HA-tagged VDAC2 in HONE1 and SUNE1 cells. **i**, WT and TRIM21-KO1 HONE1 and SUNE1 cells were treated with DMSO or DIDS (100μm) for 24 h, and then received IR (6Gy). The cytosolic mtDNA accumulation was evaluated by quantification of cytoplasmic TFAM (as indicated by the white arrows) 24 h after IR. Representative images and statistical results from 30 cells in each condition were reported (scale bar, 10 μm). The results are representative of three independent experiments (**a**-**i**) and are shown as the mean±SEM (**a-c, e, g-i**). Statistical analysis was performed by one-way ANOVA with the Tukey's test for multiple comparisons.

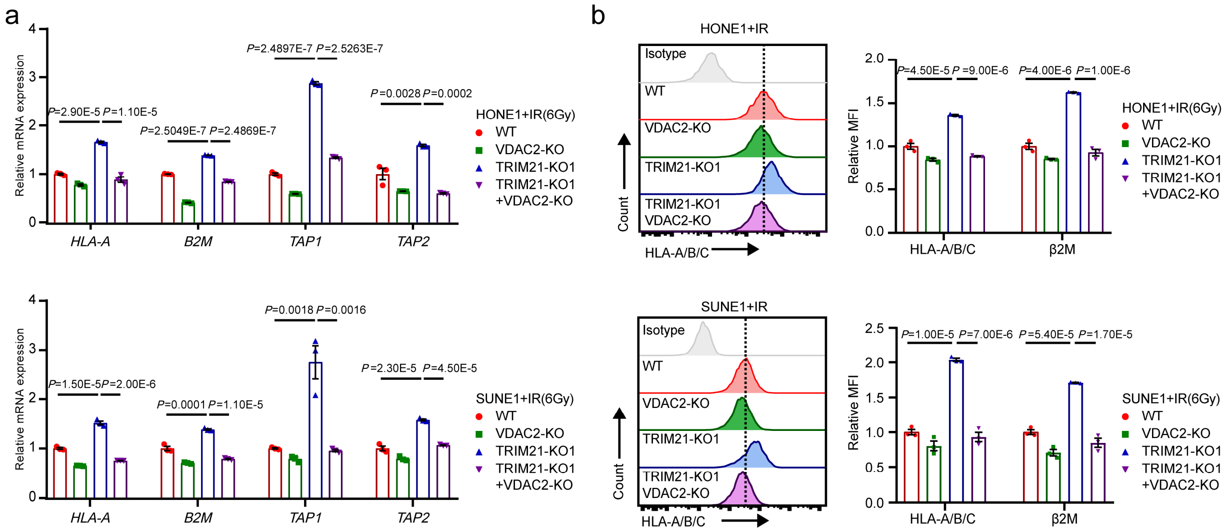

Supplementary Fig. 6. (Extended data related to Main Fig. 5)

a, Relative *HLA-A*, *B2M*, *TAP1*, and *TAP2* mRNA expression in WT, VDAC2-KO, TRIM21-KO, and TRIM21-KO+ VDAC2-KO HONE1 and SUNE1 cells 48 h after IR treatment. **b,** Flow cytometric analysis of surface HLA-A/B/C and β2M expression on WT, VDAC2-KO, TRIM21-KO, and TRIM21-KO+ VDAC2-KO HONE1 and SUNE1 cells 48 h after IR treatment. The data are shown as the mean±SEM of three independent experiments. Statistical analysis was performed by one-way ANOVA with the Tukey's test for multiple comparisons.

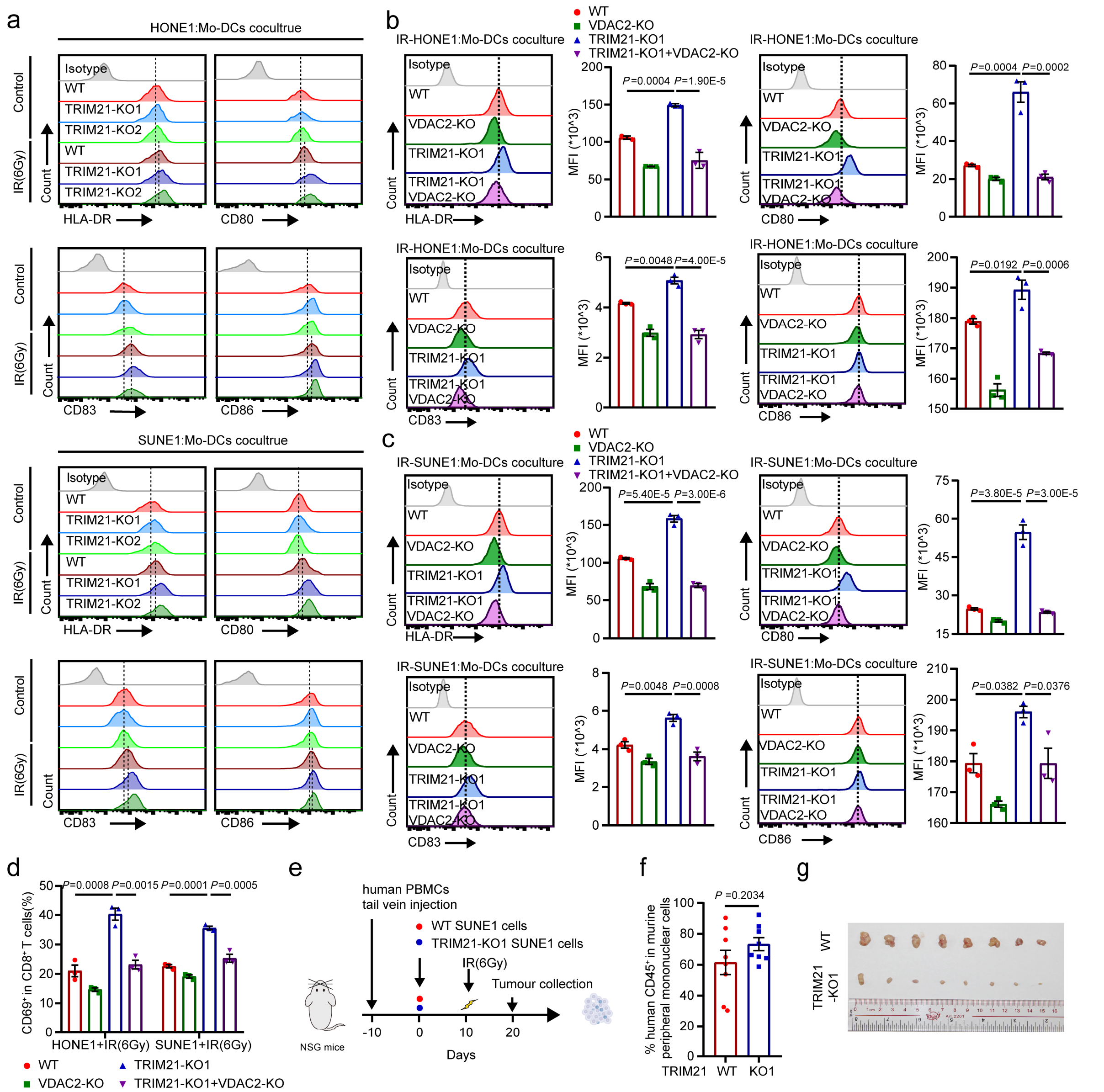

Supplementary Fig. 7. (Extended data related to Main Fig. 6)

a, Representative histogram of the expression of maturation markers, including HLA-DR, CD80, CD83, and CD86, on Mo-DCs cocultured with WT or *TRIM21*-KO HONE1 or SUNE1 cells. **b-c,** WT, VDAC2-KO, TRIM21-KO, and TRIM21-KO+ VDAC2-KO NPC cells were treated with IR and were then cocultured with Mo-DCs for another 48 h. The expression of maturation markers, including HLA-DR, CD80, CD83, and CD86, on Mo-DCs was measured by flow cytometry. **d,** Irradiated WT, VDAC2-KO, TRIM21-KO, and TRIM21-KO+ VDAC2-KO NPC cells were cocultured with peripheral blood monocytes (PBMCs) for 24 h. The proportion of $CD69^+$ cells among $CD8^+$ T cells was determined by flow cytometry. **e,** Five-week-old female NSG mice were injected with 5×10^6 human PBMCs via the tail vein. Ten days after injection of PBMCs, 1×10^6 WT or TRIM21-KO SUNE1 cells were injected subcutaneously. Tumours were treated with IR on day 10 after cell inoculation and were then harvested at the indicated time. **f,** Percentage of human $CD45^+$ cells among peripheral mononuclear cells in humanized NSG mice (n = 8 in each group). **g,** Macroscopic images of the excised residual tumours in each group. n = 3 independent experiments in **b-d.** Data in **b-d,** and **f** are shown as the mean±SEM. Statistical analysis was performed by one-way ANOVA with the Tukey's test for multiple comparisons in **b-d**, and by a two-tailed unpaired t test in **f**.

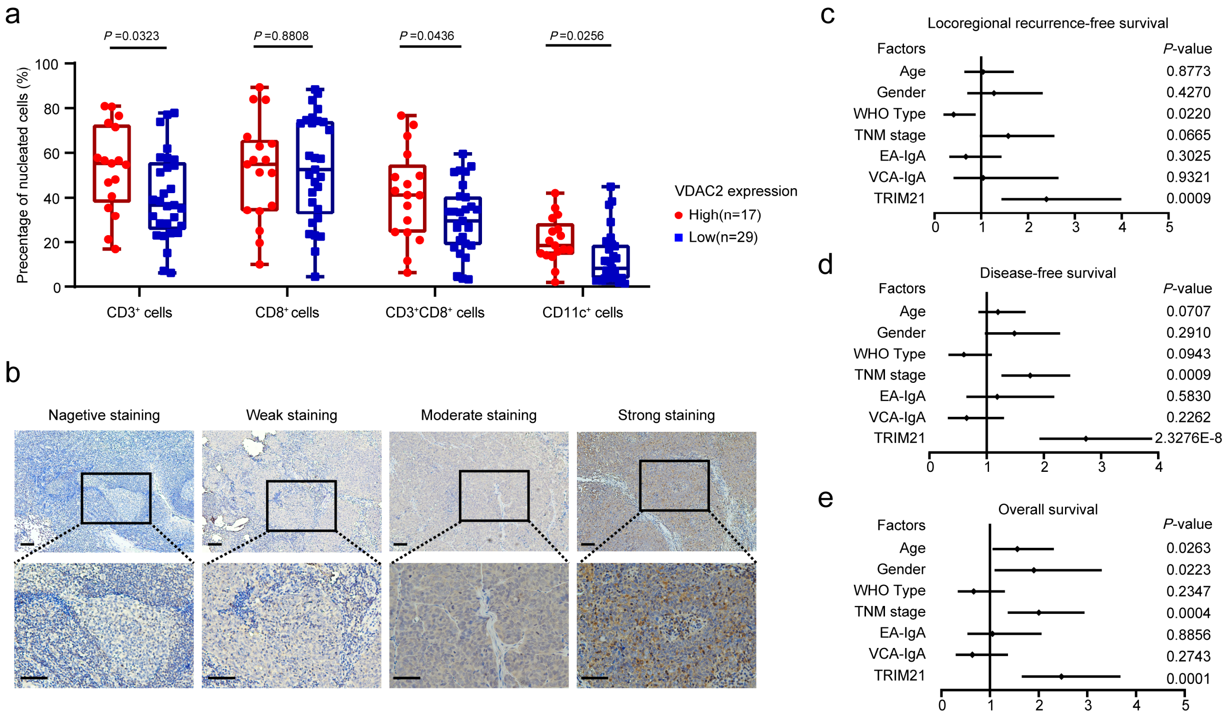

Supplementary Fig. 8. (Extended data related to Main Fig. 7)

a, Quantitative results of multiplex immunohistochemistry for $CD3^+$, $CD8^+$, $CD3^+CD8^+$ and $CD11c^+$ cells in NPC tumours with high or low VDAC2 expression (the data are presented as the means ± SEMs; two-tailed unpaired t test). **b**, Representative images of IHC staining for TRIM21 in NPC tissues scored according to the staining intensity (scale bar, 50 µm). **c-e**, Forest plots showing the results of multivariate Cox regression analysis indicated that TRIM21 expression is a significant prognostic indicator for locoregional recurrence-free (**c**), disease-free (**d**) and overall (**e**) survival in NPC patients (Cox proportional-hazards model).

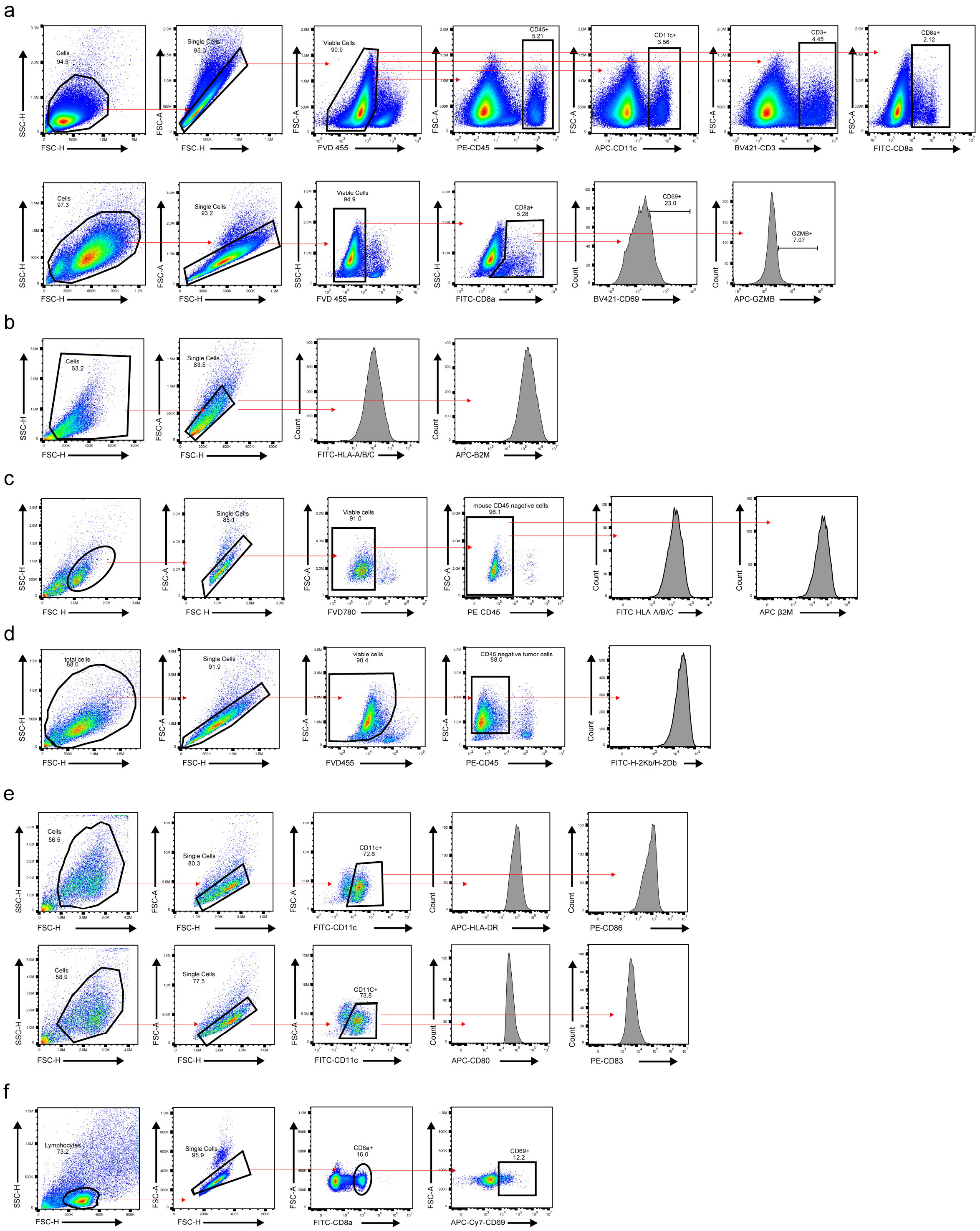

Supplementary Fig. 9 (Gating strategy for flow cytometric analysis)

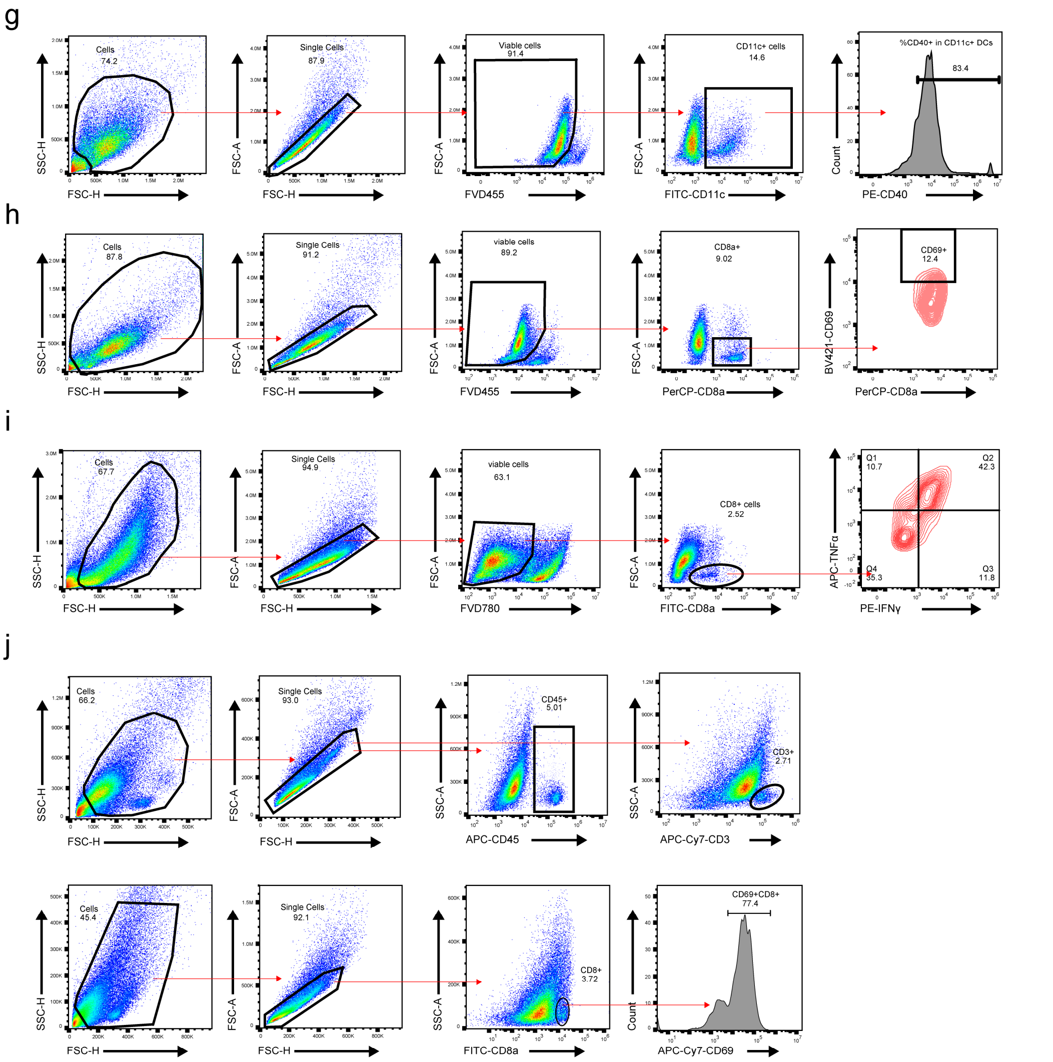

Supplementary Fig. 9 (Gating strategy for flow cytometric analysis)

a, Gating strategy for the flow cytometric analysis in Fig. 1m and Supplementary Fig. 1j and 1k.

b, Gating strategy for the flow cytometric analysis in Fig. 5b and Supplementary Fig. 6b.

c, Gating strategy for the flow cytometric analysis in Fig. 5f.

d, Gating strategy for the flow cytometric analysis in Fig. 5g.

e, Gating strategy for the flow cytometric analysis in Fig. 6a and Supplementary Fig. 7a-c.

f, Gating strategy for the flow cytometric analysis in Fig. 6b and Supplementary Fig. 7d.

g, Gating strategy for the flow cytometric analysis in Fig. 6d.

h, Gating strategy for the flow cytometric analysis in Fig. 6e.

i, Gating strategy for the flow cytometric analysis in Fig. 6f.

j, Gating strategy for the flow cytometric analysis in Fig. 6i.

Supplementary Table 1. Mass spectrometry analysis results for the anti-FLAG (FLAG-tagged TRIM21) immunoprecipitate.

Accession	-10lgP	Area	#Peptides	Description
sp|P19474|RO52_HUMAN	311.56	6.93E+09	58	E3 ubiquitin-protein ligase TRIM21 OS=Homo sapiens OX=9606 GN=TRIM21 PE=1 SV=1
sp|Q9UHB6|LIMA1_HUMAN	311.26	2.27E+09	52	LIM domain and actin-binding protein 1 OS=Homo sapiens OX=9606 GN=LIMA1 PE=1 SV=1
sp|P04264|K2C1_HUMAN	301.06	9.43E+08	50	Keratin type II cytoskeletal 1 OS=Homo sapiens OX=9606 GN=KRT1 PE=1 SV=6
sp|P13645|K1C10_HUMAN	297.4	1.69E+09	43	Keratin type I cytoskeletal 10 OS=Homo sapiens OX=9606 GN=KRT10 PE=1 SV=6
sp|P35908|K22E_HUMAN	296.45	6.04E+06	45	Keratin type II cytoskeletal 2 epidermal OS=Homo sapiens OX=9606 GN=KRT2 PE=1 SV=2
sp|P35527|K1C9_HUMAN	283.48	5.60E+08	35	Keratin type I cytoskeletal 9 OS=Homo sapiens OX=9606 GN=KRT9 PE=1 SV=3
sp|P08670|VIME_HUMAN	279.82	5.23E+08	46	Vimentin OS=Homo sapiens OX=9606 GN=VIM PE=1 SV=4
sp|P60709|ACTB_HUMAN	265.34	1.17E+07	33	Actin cytoplasmic 1 OS=Homo sapiens OX=9606 GN=ACTB PE=1 SV=1
sp|P63261|ACTG_HUMAN	264.7	5.34E+06	33	Actin cytoplasmic 2 OS=Homo sapiens OX=9606 GN=ACTG1 PE=1 SV=1
sp|P05787|K2C8_HUMAN	256.13	1.08E+08	41	Keratin type II cytoskeletal 8 OS=Homo sapiens OX=9606 GN=KRT8 PE=1 SV=7
sp|P07355|ANXA2_HUMAN	255.11	3.34E+07	28	Annexin A2 OS=Homo sapiens OX=9606 GN=ANXA2 PE=1 SV=2
sp|Q8IVT2|MISP_HUMAN	250.8	2.16E+08	29	Mitotic interactor and substrate of PLK1 OS=Homo sapiens OX=9606 GN=MISP PE=1 SV=1
sp|P07437|TBB5_HUMAN	248.62	3.12E+07	25	Tubulin beta chain OS=Homo sapiens OX=9606 GN=TUBB PE=1 SV=2
sp|O15231|ZN185_HUMAN	245.44	2.06E+08	32	Zinc finger protein 185 OS=Homo sapiens OX=9606 GN=ZNF185 PE=1 SV=3
sp|P68371|TBB4B_HUMAN	242.79	1.31E+07	24	Tubulin beta-4B chain OS=Homo sapiens OX=9606 GN=TUBB4B PE=1 SV=1
sp|P08729|K2C7_HUMAN	236.86	9.78E+06	36	Keratin type II cytoskeletal 7 OS=Homo sapiens OX=9606 GN=KRT7 PE=1 SV=5
sp|P68133|ACTS_HUMAN	223.36	9.05E+07	22	Actin alpha skeletal muscle OS=Homo sapiens OX=9606 GN=ACTA1 PE=1 SV=1
sp|P68032|ACTC_HUMAN	223.36	9.05E+07	22	Actin alpha cardiac muscle 1 OS=Homo sapiens OX=9606 GN=ACTC1 PE=1 SV=1
sp|Q5VTE0|EF1A3_HUMAN	218.19	1.36E+08	19	Putative elongation factor 1-alpha-like 3 OS=Homo sapiens OX=9606 GN=EEF1A1P5 PE=5 SV=1
sp|P02538|K2C6A_HUMAN	217.92	2.32E+06	27	Keratin type II cytoskeletal 6A OS=Homo sapiens OX=9606 GN=KRT6A PE=1 SV=3
sp|P02533|K1C14_HUMAN	217.74	1.73E+07	29	Keratin type I cytoskeletal 14 OS=Homo sapiens OX=9606 GN=KRT14 PE=1 SV=4
sp|Q9BQE3|TBA1C_HUMAN	217.67	6.46E+05	17	Tubulin alpha-1C chain OS=Homo sapiens OX=9606 GN=TUBA1C PE=1 SV=1
sp|P52907|CAZA1_HUMAN	215.61	1.21E+08	17	F-actin-capping protein subunit alpha-1 OS=Homo sapiens OX=9606 GN=CAPZA1 PE=1 SV=3
sp|Q9ULV4|COR1C_HUMAN	210.73	5.00E+07	20	Coronin-1C OS=Homo sapiens OX=9606 GN=CORO1C PE=1 SV=1
sp|Q04695|K1C17_HUMAN	205.48	1.39E+07	25	Keratin type I cytoskeletal 17 OS=Homo sapiens OX=9606 GN=KRT17 PE=1 SV=2
sp|P13647|K2C5_HUMAN	201.59	1.55E+07	29	Keratin type II cytoskeletal 5 OS=Homo sapiens OX=9606 GN=KRT5 PE=1 SV=3
sp|P68366|TBA4A_HUMAN	199.42	1.55E+06	15	Tubulin alpha-4A chain OS=Homo sapiens OX=9606 GN=TUBA4A PE=1 SV=1
sp|P35579|MYH9_HUMAN	195.69	1.04E+07	18	Myosin-9 OS=Homo sapiens OX=9606 GN=MYH9 PE=1 SV=4
sp|P67936|TPM4_HUMAN	191.95	3.91E+07	16	Tropomyosin alpha-4 chain OS=Homo sapiens OX=9606 GN=TPM4 PE=1 SV=3
sp|P36873|PP1G_HUMAN	187.86	3.71E+06	13	Serine/threonine-protein phosphatase PP1-gamma catalytic subunit OS=Homo sapiens OX=9606 GN=PPP1CC PE=1 SV=1

sp|O43707|ACTN4_HUMAN	184.1	1.22E+07	15	Alpha-actinin-4 OS=Homo sapiens OX=9606 GN=ACTN4 PE=1 SV=2
sp|P62136|PP1A_HUMAN	183.36	6.88E+05	12	Serine/threonine-protein phosphatase PP1-alpha catalytic subunit OS=Homo sapiens OX=9606 GN=PPP1CA PE=1 SV=1
sp|P08238|HS90B_HUMAN	180.31	1.20E+07	14	Heat shock protein HSP 90-beta OS=Homo sapiens OX=9606 GN=HSP90AB1 PE=1 SV=4
sp|P04406|G3P_HUMAN	179.8	1.16E+08	11	Glyceraldehyde-3-phosphate dehydrogenase OS=Homo sapiens OX=9606 GN=GAPDH PE=1 SV=3
sp|P62140|PP1B_HUMAN	175.54	2.60E+05	10	Serine/threonine-protein phosphatase PP1-beta catalytic subunit OS=Homo sapiens OX=9606 GN=PPP1CB PE=1 SV=3
sp|P08779|K1C16_HUMAN	175.08	2.38E+06	18	Keratin type I cytoskeletal 16 OS=Homo sapiens OX=9606 GN=KRT16 PE=1 SV=4
sp|Q562R1|ACTBL_HUMAN	173.65	0.00E+00	13	Beta-actin-like protein 2 OS=Homo sapiens OX=9606 GN=ACTBL2 PE=1 SV=2
sp|P47755|CAZA2_HUMAN	171.97	2.38E+07	12	F-actin-capping protein subunit alpha-2 OS=Homo sapiens OX=9606 GN=CAPZA2 PE=1 SV=3
sp|P06753|TPM3_HUMAN	169.73	4.71E+07	11	Tropomyosin alpha-3 chain OS=Homo sapiens OX=9606 GN=TPM3 PE=1 SV=2
sp|P12814|ACTN1_HUMAN	157.32	1.19E+06	10	Alpha-actinin-1 OS=Homo sapiens OX=9606 GN=ACTN1 PE=1 SV=2
sp|Q12792|TWF1_HUMAN	150.96	1.12E+07	7	Twinfilin-1 OS=Homo sapiens OX=9606 GN=TWF1 PE=1 SV=3
sp|Q15149|PLEC_HUMAN	141.95	2.60E+07	10	Plectin OS=Homo sapiens OX=9606 GN=PLEC PE=1 SV=3
sp|P20592|MX2_HUMAN	135.74	1.05E+07	9	Interferon-induced GTP-binding protein Mx2 OS=Homo sapiens OX=9606 GN=MX2 PE=1 SV=1
sp|O14639|ABLM1_HUMAN	133.86	1.36E+07	7	Actin-binding LIM protein 1 OS=Homo sapiens OX=9606 GN=ABLIM1 PE=1 SV=3
sp|P04899|GNAI2_HUMAN	132.93	5.17E+06	8	Guanine nucleotide-binding protein G(i) subunit alpha-2 OS=Homo sapiens OX=9606 GN=GNAI2 PE=1 SV=3
sp|Q96C19|EFHD2_HUMAN	131.19	8.34E+06	7	EF-hand domain-containing protein D2 OS=Homo sapiens OX=9606 GN=EFHD2 PE=1 SV=1
sp|P08754|GNAI3_HUMAN	127.29	3.01E+06	7	Guanine nucleotide-binding protein G(i) subunit alpha OS=Homo sapiens OX=9606 GN=GNAI3 PE=1 SV=3
sp|Q7Z3Y7|K1C28_HUMAN	118.43	2.10E+07	5	Keratin type I cytoskeletal 28 OS=Homo sapiens OX=9606 GN=KRT28 PE=1 SV=2
sp|P07900|HS90A_HUMAN	115.53	3.33E+05	6	Heat shock protein HSP 90-alpha OS=Homo sapiens OX=9606 GN=HSP90AA1 PE=1 SV=5
sp|P62987|RL40_HUMAN	115.31	1.16E+07	5	Ubiquitin-60S ribosomal protein L40 OS=Homo sapiens OX=9606 GN=UBA52 PE=1 SV=2
sp|P62979|RS27A_HUMAN	115.31	1.16E+07	5	Ubiquitin-40S ribosomal protein S27a OS=Homo sapiens OX=9606 GN=RPS27A PE=1 SV=2
sp|P0CG47|UBB_HUMAN	115.31	1.16E+07	5	Polyubiquitin-B OS=Homo sapiens OX=9606 GN=UBB PE=1 SV=1
sp|P0CG48|UBC_HUMAN	115.31	1.16E+07	5	Polyubiquitin-C OS=Homo sapiens OX=9606 GN=UBC PE=1 SV=3
sp|P45880|VDAC2_HUMAN	114.89	7.40E+06	5	Voltage-dependent anion-selective channel protein 2 OS=Homo sapiens OX=9606 GN=VDAC2 PE=1 SV=2
sp|P35580|MYH10_HUMAN	114.76	7.14E+05	6	Myosin-10 OS=Homo sapiens OX=9606 GN=MYH10 PE=1 SV=3
sp|P62873|GBB1_HUMAN	111.23	5.78E+06	6	Guanine nucleotide-binding protein G(I)/G(S)/G(T) subunit beta-1 OS=Homo sapiens OX=9606 GN=GNB1 PE=1 SV=3
sp|P80723|BASP1_HUMAN	109.52	8.69E+06	4	Brain acid soluble protein 1 OS=Homo sapiens OX=9606 GN=BASP1 PE=1 SV=2
sp|Q16643|DREB_HUMAN	108.29	1.84E+07	6	Drebrin OS=Homo sapiens OX=9606 GN=DBN1 PE=1 SV=4
sp|Q86YZ3|HORN_HUMAN	106.98	4.62E+06	3	Hornerin OS=Homo sapiens OX=9606 GN=HRNR PE=1 SV=2
sp|Q2M2I5|K1C24_HUMAN	103.77	3.13E+06	6	Keratin type I cytoskeletal 24 OS=Homo sapiens OX=9606 GN=KRT24 PE=1 SV=1
sp|P41219|PERI_HUMAN	103.51	5.09E+05	4	Peripherin OS=Homo sapiens OX=9606 GN=PRPH PE=1 SV=2
sp|P81605|DCD_HUMAN	102.09	9.87E+06	4	Dermcidin OS=Homo sapiens OX=9606 GN=DCD PE=1 SV=2

sp|Q8NHW5|RLA0L_HUMAN	101.78	2.39E+06	3	60S acidic ribosomal protein P0-like OS=Homo sapiens OX=9606 GN=RPLP0P6 PE=5 SV=1
sp|P05388|RLA0_HUMAN	101.78	2.39E+06	3	60S acidic ribosomal protein P0 OS=Homo sapiens OX=9606 GN=RPLP0 PE=1 SV=1
sp|P14136|GFAP_HUMAN	100.84	1.15E+06	5	Glial fibrillary acidic protein OS=Homo sapiens OX=9606 GN=GFAP PE=1 SV=1
sp|P05783|K1C18_HUMAN	100.61	4.03E+06	7	Keratin type I cytoskeletal 18 OS=Homo sapiens OX=9606 GN=KRT18 PE=1 SV=2
sp|Q5T749|KPRP_HUMAN	99.94	9.35E+06	5	Keratinocyte proline-rich protein OS=Homo sapiens OX=9606 GN=KPRP PE=1 SV=1
sp|P62879|GBB2_HUMAN	98.92	3.83E+06	4	Guanine nucleotide-binding protein G(I)/G(S)/G(T) subunit beta-2 OS=Homo sapiens OX=9606 GN=GNB2 PE=1 SV=3
sp|Q9NSB2|KRT84_HUMAN	98.07	3.17E+06	5	Keratin type II cuticular Hb4 OS=Homo sapiens OX=9606 GN=KRT84 PE=2 SV=2
sp|Q9NR12|PDLI7_HUMAN	89.63	3.79E+06	5	PDZ and LIM domain protein 7 OS=Homo sapiens OX=9606 GN=PDLIM7 PE=1 SV=1
sp|P15880|RS2_HUMAN	88.83	2.87E+06	2	40S ribosomal protein S2 OS=Homo sapiens OX=9606 GN=RPS2 PE=1 SV=2
sp|P31943|HNRH1_HUMAN	88.23	4.04E+06	7	Heterogeneous nuclear ribonucleoprotein H OS=Homo sapiens OX=9606 GN=HNRNPH1 PE=1 SV=4
sp|A2NJV5|KV229_HUMAN	84.06	3.24E+07	2	Immunoglobulin kappa variable 2-29 OS=Homo sapiens OX=9606 GN=IGKV2-29 PE=3 SV=2
sp|A0A0A0MRZ7|KVD26_HUMAN	84.06	3.24E+07	2	Immunoglobulin kappa variable 2D-26 OS=Homo sapiens OX=9606 GN=IGKV2D-26 PE=3 SV=1
sp|A0A075B6S2|KVD29_HUMAN	84.06	3.24E+07	2	Immunoglobulin kappa variable 2D-29 OS=Homo sapiens OX=9606 GN=IGKV2D-29 PE=3 SV=1
sp|O00214|LEG8_HUMAN	82.03	1.97E+06	3	Galectin-8 OS=Homo sapiens OX=9606 GN=LGALS8 PE=1 SV=4
sp|P25705|ATPA_HUMAN	81.97	2.17E+06	5	ATP synthase subunit alpha mitochondrial OS=Homo sapiens OX=9606 GN=ATP5F1A PE=1 SV=1
sp|P61247|RS3A_HUMAN	79.13	4.07E+06	4	40S ribosomal protein S3a OS=Homo sapiens OX=9606 GN=RPS3A PE=1 SV=2
sp|P14923|PLAK_HUMAN	74.7	1.61E+06	3	Junction plakoglobin OS=Homo sapiens OX=9606 GN=JUP PE=1 SV=3
sp|P01859|IGHG2_HUMAN	74.04	8.79E+06	2	Immunoglobulin heavy constant gamma 2 OS=Homo sapiens OX=9606 GN=IGHG2 PE=1 SV=2
sp|P07196|NFL_HUMAN	66.06	7.45E+05	2	Neurofilament light polypeptide OS=Homo sapiens OX=9606 GN=NEFL PE=1 SV=3
sp|P33993|MCM7_HUMAN	65.92	1.64E+06	3	DNA replication licensing factor MCM7 OS=Homo sapiens OX=9606 GN=MCM7 PE=1 SV=4
sp|Q01082|SPTB2_HUMAN	62.36	1.50E+06	4	Spectrin beta chain non-erythrocytic 1 OS=Homo sapiens OX=9606 GN=SPTBN1 PE=1 SV=2
sp|P07195|LDHB_HUMAN	61.53	1.61E+06	2	L-lactate dehydrogenase B chain OS=Homo sapiens OX=9606 GN=LDHB PE=1 SV=2
sp|Q15366|PCBP2_HUMAN	60.56	1.78E+06	2	Poly(rC)-binding protein 2 OS=Homo sapiens OX=9606 GN=PCBP2 PE=1 SV=1
sp|Q8WWI1|LMO7_HUMAN	58.19	1.01E+06	4	LIM domain only protein 7 OS=Homo sapiens OX=9606 GN=LMO7 PE=1 SV=3
sp|Q9Y608|LRRF2_HUMAN	57.12	1.15E+06	2	Leucine-rich repeat flightless-interacting protein 2 OS=Homo sapiens OX=9606 GN=LRRFIP2 PE=1 SV=1
sp|P06576|ATPB_HUMAN	56.2	7.53E+05	1	ATP synthase subunit beta mitochondrial OS=Homo sapiens OX=9606 GN=ATP5F1B PE=1 SV=3
sp|P07910|HNRPC_HUMAN	55.77	2.59E+06	2	Heterogeneous nuclear ribonucleoproteins C1/C2 OS=Homo sapiens OX=9606 GN=HNRNPC PE=1 SV=4
sp|P05109|S10A8_HUMAN	55.16	4.94E+05	2	Protein S100-A8 OS=Homo sapiens OX=9606 GN=S100A8 PE=1 SV=1
sp|Q4KMQ1|TPRN_HUMAN	54.2	2.04E+05	1	Taperin OS=Homo sapiens OX=9606 GN=TPRN PE=1 SV=2
sp|Q5D862|FILA2_HUMAN	54.07	1.64E+06	1	Filaggrin-2 OS=Homo sapiens OX=9606 GN=FLG2 PE=1 SV=1
sp|O00299|CLIC1_HUMAN	53.57	9.95E+05	2	Chloride intracellular channel protein 1 OS=Homo sapiens OX=9606 GN=CLIC1 PE=1 SV=4
sp|Q5T750|XP32_HUMAN	49.56	2.68E+06	1	Skin-specific protein 32 OS=Homo sapiens OX=9606 GN=XP32 PE=1 SV=1

sp|Q9BUP3|HTAI2_HUMAN	49.35	5.98E+06	2	Oxidoreductase HTATIP2 OS=Homo sapiens OX=9606 GN=HTATIP2 PE=1 SV=2
sp|P26641|EF1G_HUMAN	49.1	7.91E+05	2	Elongation factor 1-gamma OS=Homo sapiens OX=9606 GN=EEF1G PE=1 SV=3
sp|P62891|RL39_HUMAN	48.35	3.82E+06	1	60S ribosomal protein L39 OS=Homo sapiens OX=9606 GN=RPL39 PE=1 SV=2
sp|Q59GN2|R39L5_HUMAN	48.35	3.82E+06	1	Putative 60S ribosomal protein L39-like 5 OS=Homo sapiens OX=9606 GN=RPL39P5 PE=5 SV=2
sp|A0A0B4J1V7|HV781_HUMAN	46.5	6.91E+07	2	Probable non-functional immunoglobulin heavy variable 7-81 OS=Homo sapiens OX=9606 GN=IGHV7-81 PE=5 SV=1
sp|P15924|DESP_HUMAN	45.56	8.89E+05	2	Desmoplakin OS=Homo sapiens OX=9606 GN=DSP PE=1 SV=3
sp|P36542|ATPG_HUMAN	41.73	3.34E+05	1	ATP synthase subunit gamma mitochondrial OS=Homo sapiens OX=9606 GN=ATP5F1C PE=1 SV=1
sp|P62753|RS6_HUMAN	40.6	1.35E+06	1	40S ribosomal protein S6 OS=Homo sapiens OX=9606 GN=RPS6 PE=1 SV=1
sp|Q07021|C1QBP_HUMAN	37.41	2.31E+05	1	Complement component 1 Q subcomponent-binding protein mitochondrial OS=Homo sapiens OX=9606 GN=C1QBP PE=1 SV=1
sp|P46777|RL5_HUMAN	37.23	3.91E+05	1	60S ribosomal protein L5 OS=Homo sapiens OX=9606 GN=RPL5 PE=1 SV=3
sp|P21980|TGM2_HUMAN	37.08	6.36E+05	2	Protein-glutamine gamma-glutamyltransferase 2 OS=Homo sapiens OX=9606 GN=TGM2 PE=1 SV=2
sp|Q0ZGT2|NEXN_HUMAN	34.26	1.02E+06	1	Nexilin OS=Homo sapiens OX=9606 GN=NEXN PE=1 SV=1
sp|P62917|RL8_HUMAN	34.21	5.52E+05	1	60S ribosomal protein L8 OS=Homo sapiens OX=9606 GN=RPL8 PE=1 SV=2
sp|Q5TH69|BIG3_HUMAN	34.05	2.89E+07	2	Brefeldin A-inhibited guanine nucleotide-exchange protein 3 OS=Homo sapiens OX=9606 GN=ARFGEF3 PE=1 SV=3
sp|P14618|KPYM_HUMAN	32.14	5.84E+05	1	Pyruvate kinase PKM OS=Homo sapiens OX=9606 GN=PKM PE=1 SV=4
sp|P16403|H12_HUMAN	30.63	5.48E+05	1	Histone H1.2 OS=Homo sapiens OX=9606 GN=H1-2 PE=1 SV=2
sp|P10412|H14_HUMAN	30.63	5.48E+05	1	Histone H1.4 OS=Homo sapiens OX=9606 GN=H1-4 PE=1 SV=2
sp|P16402|H13_HUMAN	30.63	5.48E+05	1	Histone H1.3 OS=Homo sapiens OX=9606 GN=H1-3 PE=1 SV=2
sp|P36578|RL4_HUMAN	30.61	4.82E+05	1	60S ribosomal protein L4 OS=Homo sapiens OX=9606 GN=RPL4 PE=1 SV=5
sp|Q8N442|GUF1_HUMAN	30.37	7.28E+06	1	Translation factor GUF1 mitochondrial OS=Homo sapiens OX=9606 GN=GUF1 PE=1 SV=1
sp|Q9P0M6|H2AW_HUMAN	29.6	1.63E+05	1	Core histone macro-H2A.2 OS=Homo sapiens OX=9606 GN=MACROH2A2 PE=1 SV=3
sp|P29692|EF1D_HUMAN	29.25	4.95E+05	1	Elongation factor 1-delta OS=Homo sapiens OX=9606 GN=EEF1D PE=1 SV=5
sp|Q8WXE9|STON2_HUMAN	27.39	6.92E+06	1	Stonin-2 OS=Homo sapiens OX=9606 GN=STON2 PE=1 SV=1
sp|P06733|ENOA_HUMAN	26.59	5.50E+05	1	Alpha-enolase OS=Homo sapiens OX=9606 GN=ENO1 PE=1 SV=2
sp|Q6P3X8|PGBD2_HUMAN	25.97	2.56E+06	1	PiggyBac transposable element-derived protein 2 OS=Homo sapiens OX=9606 GN=PGBD2 PE=2 SV=1
sp|O76041|NEBL_HUMAN	25.3	4.64E+05	1	Nebulette OS=Homo sapiens OX=9606 GN=NEBL PE=1 SV=1
sp|Q12851|M4K2_HUMAN	24.74	1.08E+07	1	Mitogen-activated protein kinase kinase kinase kinase 2 OS=Homo sapiens OX=9606 GN=MAP4K2 PE=1 SV=2
sp|Q14689|DIP2A_HUMAN	24.74	1.08E+07	1	Disco-interacting protein 2 homolog A OS=Homo sapiens OX=9606 GN=DIP2A PE=1 SV=2
sp|O94823|AT10B_HUMAN	24.74	1.08E+07	1	Probable phospholipid-transporting ATPase VB OS=Homo sapiens OX=9606 GN=ATP10B PE=2 SV=2
sp|O43143|DHX15_HUMAN	24.38	2.68E+05	1	Pre-mRNA-splicing factor ATP-dependent RNA helicase DHX15 OS=Homo sapiens OX=9606 GN=DHX15 PE=1 SV=2
sp|Q13813|SPTN1_HUMAN	24.34	1.34E+05	1	Spectrin alpha chain non-erythrocytic 1 OS=Homo sapiens OX=9606 GN=SPTAN1 PE=1 SV=3
sp|Q7Z6R9|AP2D_HUMAN	23.55	9.46E+05	1	Transcription factor AP-2-delta OS=Homo sapiens OX=9606 GN=TFAP2D PE=1 SV=1

sp|P06702|S10A9_HUMAN	23.42	1.33E+06	1	Protein S100-A9 OS=Homo sapiens OX=9606 GN=S100A9 PE=1 SV=1
sp|Q8N108|MIER1_HUMAN	22.94	8.60E+05	1	Mesoderm induction early response protein 1 OS=Homo sapiens OX=9606 GN=MIER1 PE=1 SV=2
sp|Q12772|SRBP2_HUMAN	22.38	5.93E+05	1	Sterol regulatory element-binding protein 2 OS=Homo sapiens OX=9606 GN=SREBF2 PE=1 SV=2
sp|Q96HC4|PDLI5_HUMAN	21.43	8.86E+05	1	PDZ and LIM domain protein 5 OS=Homo sapiens OX=9606 GN=PDLIM5 PE=1 SV=5
sp|Q14573|ITPR3_HUMAN	21.41	2.31E+05	1	Inositol 1 4 5-trisphosphate receptor type 3 OS=Homo sapiens OX=9606 GN=ITPR3 PE=1 SV=2
sp|P21817|RYR1_HUMAN	21.05	9.52E+05	1	Ryanodine receptor 1 OS=Homo sapiens OX=9606 GN=RYR1 PE=1 SV=3
sp|Q9BXX3|AN30A_HUMAN	20.7	6.25E+05	1	Ankyrin repeat domain-containing protein 30A OS=Homo sapiens OX=9606 GN=ANKRD30A PE=2 SV=3
sp|Q8IUH3|RBM45_HUMAN	20.44	4.90E+06	1	RNA-binding protein 45 OS=Homo sapiens OX=9606 GN=RBM45 PE=1 SV=1
sp|O15405|TOX3_HUMAN	20.11	1.82E+07	1	TOX high mobility group box family member 3 OS=Homo sapiens OX=9606 GN=TOX3 PE=1 SV=2

Supplementary Table 2. Mass spectrometry analysis results for the ubiquitination site in the anti-FLAG (FLAG-tagged VDAC2) immunoprecipitate.

Confidence	Sequence	Modification	Quality PEP	Quality q-value	Master protein accession code
High	TLSDYNIQKESTLHLVLR	1xGG [K9]	2.18E-05	0.00022	P62987
High	LIFAGKQLEDGR	1xGG [K6]	0.000311	0.00022	P62987
High	TLTGKTITLEVEPSDTIENVK	1xGG [K5]	0.001435	0.00022	P62987
High	NNHETQFGINHFAGIVYYETQGFLEK	1xGG [K26]	0.193923	0.009972	Q13402
High	ESEQDVEDSQNHTGEPVGDDYK	1xGG [K22]	0.106818	0.004855	Q8N8J7
High	GQTALGFLYASGLGVNSSQAK	1xGG [K21]	0.0676	0.002096	Q9UBV2
High	LTFDTTFSPNTGKK	1xGG [K13]	0.088048	0.003356	P45880
High	QSLDLFLPDK	1xGG [K10]	0.055056	0.00158	Q9NWS8

Supplementary Table 3. Correlations between TRIM21 expression and the clinical characteristics of patients with locoregionally advanced NPC.

Characteristic	Low expression group n=274(100%)	High expression group n=81(100%)	*P* value*
Age			
≤45	137 (50.0)	38 (46.9)	0.625
>45	137 (50.0)	43 (53.1)	
Gender			
Male	203 (74.1)	65 (80.2)	0.258
Female	71 (25.9)	16 (19.8)	
WHO type			
IIa	8 (2.9)	10 (12.3)	0.001
IIb	266 (97.1)	71 (87.7)	
TNM stage			
III	156 (56.9)	45 (55.6)	0.826
IV	118 (43.1)	36 (44.4)	
VCA-IgA titre			
<1:80	39 (14.2)	11 (13.6)	0.882
≥1:80	235 (85.8)	70 (86.4)	
EA-IgA titre			
<1:10	55 (20.1)	16 (19.8)	0.950
≥1:10	219 (79.9)	65 (80.2)	
Locoregional recurrence			
Yes	42 (15.3)	25 (30.9)	0.002
No	232 (84.7)	56 (69.1)	
Disease			
Yes	87 (31.8)	54 (66.7)	1.6818E-8
No	187 (68.2)	27 (33.3)	
Death			
Yes	66 (24.1)	42 (51.9)	2.00E-6
No	208 (75.9)	39 (48.1)	

*Two-sided Chi-square test. Abbreviations: WHO type IIa, differentiated nonkeratinizing NPC; WHO type IIb, undifferentiated nonkeratinizing NPC; VCA–IgA, viral capsid antigen immunoglobulin A; EA–IgA, early antigen immunoglobulin A. All patients were restaged according to the AJCC Cancer Staging Manual, 7th edition.

Supplementary Table 4. Sequences of the sgRNAs and primers used in the study.

	Sequence (forward: 5’ to 3’)
Human sgRNAs	
TRIM21 #1	GAGCCTGTGAGCATCGAGTG
TRIM21 #2	AGCACGCTTGACAATGATGT
TRIM21 #3	CAATCGACAGCTAGCCAACA
VDAC1 #1	ACTGAGACCACCAAAGTGAC
VDAC1 #2	ACTAGGCACCGAGATTACTG
VDAC1 #3	AAGCGGGAGCACATTAACCT
VDAC2 #1	AAGCAGCACAGCATTGGCCG
VDAC2 #2	TAATGTGACTCTCAAGTCCT
VDAC2 #3	AATCAAGTCTTCTTACAAGA
Human shRNA	
STING	GCAGAGCTATTTCCTTCCACA
VDAC3	CGACAGTGTAGCGTCATGTTA
Mouse sgRNA	
TRIM21 #1	GAAGCTTCACCTATTCTGTG
TRIM21 #2	GAGCCTATGAGTATCGAATG
TRIM21 #3	TAGACATATAGCCAACATGG
Primers used for RT–qPCR	
GAPDH-F	AACGGATTTGGTCGTATTGG
GAPDH-R	TTGATTTTGGAGGGATCTCG
IFNB1-F	AGGACAGGATGAACTTTGAC
IFNB1-R	TGATAGACATTAGCCAGGAG
CCL5-F	CCTGCTGCTTTGCCTACATTGC
CCL5-R	ACACACTTGGCGGTTCTTTCGG
CXCL10-F	GGTGAGAAGAGATGTCTGAATCC
CXCL10-R	GTCCATCCTTGGAAGCACTGCA
HLA-A-F	AGATACACCTGCCATGTGCAGC
HLA-A-R	GATCACAGCTCCAAGGAGAACC
B2M-F	CCACTGAAAAAGATGAGTATGCCT
B2M-R	CCAATCCAAATGCGGCATCTTCA
TAP1-F	GCAGTCAACTCCTGGACCACTA
TAP1-R	CAAGGTTCCCACTGCTTACAGC
TAP2-F	ATGCCCTTCACAATAGCAGCGG
TAP2-R	CCAAAACTGCGAACGGTCTGCA
VDAC2-F	CTTTGCAGTGGGCTACAGGACT
VDAC2-R	CGAGTGCAGTTGGTACCTGATG
Gapdh-F	CATCACTGCCACCCAGAAGACTG
Gapdh-R	ATGCCAGTGAGCTTCCCGTTCAG
Ifnb1-F	GGTGGAATGAGACTATTGTTG

Ifnb1-R	AAGTGGAGAGCAGTTGAG
Ccl5-F	CCTGCTGCTTTGCCTACCTCTC
Ccl5-R	ACACACTTGGCGGTTCCTTCGA
Cxcl10-F	ATCATCCCTGCGAGCCTATCCT
Cxcl10-R	GACCTTTTTTGGCTAAACGCTTTC
B2m-F	ACAGTTCCACCCGCCTCACATT
B2m-R	TAGAAAGACCAGTCCTTGCTGAAG
Earp1-F	CGAGGACCTGTGGAATAGCATG
Earp1-R	CATCTACAACCTCCTGACGCCA
Tap1-F	GACTCCTTGCTCTCCACTCAGT
Tap1-R	AACGCTGTCACCGTTCCAGGAT
Tap2-F	AGCAGGAAGTCAGCCGCTACAA
Tap2-R	CGCAGTTCAGAATCAGCACCTG
Tapbp-F	TGGTCAGCGTATCCAGCACTCT
Tapbp-R	TTATGGGTGAGGACGGTCAGCA
Primers used for DNA QPCR	
MTATP8(mtDNA)-F	AATATTAAACACAAACTACCACCTACC
MTATP8(mtDNA)-R	TGGTTCTCAGGGTTTGTTATA
GAPDH(gDNA)-F	CCCCACACACATGCACTTACC
GAPDH(gDNA)-R	CCTAGTCCCAGGGCTTTGATT

Supplementary Table 5. List of antibodies used in this study.

Antibodies used for Western blotting (WB) and immunoprecipitation (IP)						
**Primary antibodies**	**Supplier**	**Catalogue No.**	**Host species**	**Species activity**	**Application**	**Dilution**
anti-TRIM21	Proteintech	12108-1-AP	Rabbit	Hu, Mo	WB, IP	1:1000 for WB, 3ug for IP
anti-STING	Cell Signaling Technology	13647	Rabbit	Hu, Mo	WB	1:1000
anti-p-STING	Cell Signaling Technology	50907	Rabbit	Hu	WB	1:1000
anti-IRF3	Cell Signaling Technology	11904	Rabbit	Hu, Mo	WB	1:1000
anti-p-IRF3	Cell Signaling Technology	29047	Rabbit	Hu, Mo	WB	1:1000
anti-TBK1	Cell Signaling Technology	3504	Rabbit	Hu, Mo	WB	1:1000
anti-p-TBK1	Cell Signaling Technology	5483	Rabbit	Hu, Mo	WB	1:1000
anti-STAT1	Proteintech	10144-2-AP	Rabbit	Hu, Mo	WB	1:1000
anti-p-STAT1	Cell Signaling Technology	9167S	Rabbit	Hu, Mo	WB	1:1000
anti-VDAC1	Proteintech	55259-1-AP	Rabbit	Hu, Mo	WB	1:3000
anti-VDAC2	Proteintech	11663-1-AP	Rabbit	Hu, Mo	WB	1:1000
anti-VDAC3	Proteintech	55260-1-AP	Rabbit	Hu	WB	1:1000
anti-BAX	Proteintech	50599-2-Ig	Rabbit	Hu	WB	1:1000
anti-IFI16	Proteintech	67790-1-Ig	Mouse	Hu	WB	1:1000
anti-FLAG	Sigma–Aldrich	F3165	Mouse		WB, IP	1:1000 for WB, 3ug for IP
anti-HA	Proteintech	66006-2-Ig	Mouse		WB, IP	1:1000 for WB, 3ug for IP
anti-HA	Sigma–Aldrich	H6908	Rabbit		WB, IP	1:1000 for WB, 3ug for IP
anti-MYC	Proteintech	16286-1-AP	Rabbit		WB, IP	1:1000 for WB, 3ug for IP
anti-GAPDH	Abcam	ab8245	Mouse	Hu, Mo	WB	1:10000
anti-α-Tubulin	Abcam	66031-1-Ig	Mouse	Hu	WB	1:10000
**Secondary antibodies**						
anti-rabbit	Cell Signaling Technology	7074	Goat	rabbit	WB	1:10000
anti-mouse	Cell Signaling Technology	7076	Horse	mouse	WB	1:10000
Antibodies used for immunofluorescence (IF) and immunohistochemical (IHC) staining						
**Primary antibodies**	**Supplier**	**Catalogue No.**	**Host species**	**Species activity**	**Application**	**Dilution**
anti-dsDNA	Abcam	ab273137	Mouse	Hu	IF	1:1000
anti-FLAG	Sigma–Aldrich	F3165	Mouse		IF	1:100

anti-HA	Sigma–Aldrich	H6908	Rabbit		IF	1:100
anti-TRIM21	Proteintech	12108-1-AP	Rabbit	Hu, Mo	IHC	1:400
anti-VDAC2	Proteintech	11663-1-AP	Rabbit	Hu, Mo	IHC	1:1600
anti-CD11c	Abcam	ab52632	Rabbit	Hu	IHC	1:200
anti-CD8	Abcam	ab4055	Rabbit	Hu	IHC	1:800
anti-CD3	Abcam	ab11089	Rabbit	Hu	IHC	1:100
anti-TFAM	Proteintech	23996-1-AP	Rabbit	Hu	IF	1:200
**Secondary antibodies**						
Alexa Fluor 488	Thermo Fisher Scientific	A11008	Goat	Rabbit	IF	1:2000
Alexa Fluor 488	Thermo Fisher Scientific	A11001	Goat	Mouse	IF	1:2000
Alexa Fluor 594	Thermo Fisher Scientific	A11012	Goat	Rabbit	IF	1:2000
Antibodies used for flow cytometric analysis						
**Primary antibodies**	**Supplier**	**Catalogue**	**Clone**	**Species activity**	**Application**	**Dilution**
FITC–anti-human HLA-A/B/C	Biolegend	311404	W6/32	Hu	FC	1:100
APC–anti-human β2M	Biolegend	316312	2M2	Hu	FC	1:100
FITC–anti-human CD11c	Biolegend	337214	Bu15	Hu	FC	1:100
APC–anti-human HLA-DR	Biolegend	307610	L243	Hu	FC	1:100
APC–anti-human CD80	Biolegend	305220	2D10	Hu	FC	1:100
PE–anti-human CD83	Biolegend	305308	HB15e	Hu	FC	1:100
PE–anti-human CD86	Biolegend	374206	BU63	Hu	FC	1:100
FITC–anti-human CD8a	Biolegend	300906	HIT8a	Hu	FC	1:100
APC/Cyanine7–anti-human CD69	Biolegend	310914	FN50	Hu	FC	1:50
APC/Cyanine7–anti-human CD3	Biolegend	300425	UCHT1	Hu	FC	1:100
APC–anti-human CD45	Biolegend	304011	HI30	Hu	FC	1:100
PE–anti-mouse CD45	Biolegend	103105	30-F11	Mouse	FC	1:100
FITC–anti-mouse H-2Kb/H-2Db	Biolegend	114605	28-8-6	Mouse	FC	1:100
BV421–anti-mouse CD3	Biolegend	100227	17A2	Mouse	FC	1:100
FITC–anti-mouse CD8a	Biolegend	100706	53-6.7	Mouse	FC	1:100
PerCP–anti-mouse CD8a	Biolegend	100731	53-6.7	Mouse	FC	1:100
APC–anti-mouse CD11c	Biolegend	117309	N418	Mouse	FC	1:100
PE–anti-mouse CD40	Biolegend	124609	3/23	Mouse	FC	1:100
BV 421-anti-mouse CD69	Biolegend	104527	H1.2F3	Mouse	FC	1:50
APC–anti-human/mouse Granzyme B recombinant	Biolegend	396407	QA18A28	Mouse	ICFC	1:50
PE–anti-mouse IFNγ	Biolegend	505807	XMG1.2	Mouse	ICFC	1:50
APC–anti-mouse TNFα	Biolegend	506308	MP6-XT22	Mouse	ICFC	1:50
Antibodies used for in vivo experiment						

**Antibodies**	**Supplier**	**Catalogue**	**Clone**	**Species activity**	**Application**
InVivoMab anti-mouse CD8α	BioXcell	BE0004	NA	Mouse	in vivo CD8+ T cell depletion
InVivoMAb Rat IgG2a isotype control	BioXcell	BE0089	NA	Mouse	
InVivoMab anti-mouse IFNAR-1	BioXcell	BE0241	NA	Mouse	in vivo IFNAR-1 blockade
InVivoMAb mouse IgG1 isotype control	BioXcel	BE0083	NA	Mouse	
