# Supplementary material for: TRIM21 inhibits irradiation-induced mitochondrial DNA release and impairs antitumour immunity in nasopharyngeal carcinoma tumour models
Source: Nat Commun. 2023 Feb 16;14:865. doi: 10.1038/s41467-023-36523-y (PMC9935546; doi:10.1038/s41467-023-36523-y)
Supplement: Supplementary file 2 — Reporting Summary [file 41467_2023_36523_MOESM2_ESM.pdf]

nature portfolio

Corresponding author(s): Jun Ma

Last updated by author(s): Jan 25, 2023

Reporting Summary

Nature Portfolio wishes to improve the reproducibility of the work that we publish. This form provides structure for consistency and transparency in reporting. For further information on Nature Portfolio policies, see our Editorial Policies and the Editorial Policy Checklist.

Statistics

For all statistical analyses, confirm that the following items are present in the figure legend, table legend, main text, or Methods section.

n/a	Confirmed	
☐	☒	The exact sample size (*n*) for each experimental group/condition, given as a discrete number and unit of measurement
☐	☒	A statement on whether measurements were taken from distinct samples or whether the same sample was measured repeatedly
☐	☒	The statistical test(s) used AND whether they are one- or two-sided<br>*Only common tests should be described solely by name; describe more complex techniques in the Methods section.*
☐	☒	A description of all covariates tested
☐	☒	A description of any assumptions or corrections, such as tests of normality and adjustment for multiple comparisons
☐	☒	A full description of the statistical parameters including central tendency (e.g. means) or other basic estimates (e.g. regression coefficient) AND variation (e.g. standard deviation) or associated estimates of uncertainty (e.g. confidence intervals)
☐	☒	For null hypothesis testing, the test statistic (e.g. *F*, *t*, *r*) with confidence intervals, effect sizes, degrees of freedom and *P* value noted<br>*Give P values as exact values whenever suitable.*
☒	☐	For Bayesian analysis, information on the choice of priors and Markov chain Monte Carlo settings
☒	☐	For hierarchical and complex designs, identification of the appropriate level for tests and full reporting of outcomes
☐	☒	Estimates of effect sizes (e.g. Cohen's *d*, Pearson's *r*), indicating how they were calculated

Our web collection on statistics for biologists contains articles on many of the points above.

Software and code

Policy information about availability of computer code

Data collection	No software was used.
Data analysis	No software was used.

For manuscripts utilizing custom algorithms or software that are central to the research but not yet described in published literature, software must be made available to editors and reviewers. We strongly encourage code deposition in a community repository (e.g. GitHub). See the Nature Portfolio guidelines for submitting code & software for further information.

Data

Policy information about availability of data

All manuscripts must include a data availability statement. This statement should provide the following information, where applicable:

- Accession codes, unique identifiers, or web links for publicly available datasets
- A description of any restrictions on data availability
- For clinical datasets or third party data, please ensure that the statement adheres to our policy

The bulk and scRNA-seq data used in this study was published previously (GSE150430). All original data for this study can be obtained from the corresponding author. The authenticity of this article has been validated by uploading the key raw data onto the Research Data Deposit public platform (www.researchdata.org.cn), with the approval RDD number as RDDB2023358879.

Human research participants

Policy information about studies involving human research participants and Sex and Gender in Research.

Reporting on sex and gender	The gender information of 355 patients with nasopharyngeal carcinoma were retrospectively collected through the medical record. We reported the gender distribution of 355 NPC patients in the Supplementary Table 3 and the prognostic value of gender in the multivariate analysis (Supplementary Figure 8c-e).
Population characteristics	The patients' clinical characteristics are listed in Supplementary Table 3.
Recruitment	We collected 46 NPC samples for protein expression and tumor-infiltrating immune subpopulations analysis,and collected 355 paraffin-embedded NPC samples from the Sun Yat-sen University Cancer Center (Guangzhou, China) between January 2006 and December 2010 for survival analysis.
Ethics oversight	The Institutional Ethical Review Boards of Sun Yat-sen University Cancer Center approved this study (B2022-259-01), in which anonymized data were analysed, and waived the requirement for informed consent.

Note that full information on the approval of the study protocol must also be provided in the manuscript.

Field-specific reporting

Please select the one below that is the best fit for your research. If you are not sure, read the appropriate sections before making your selection.

- [x] Life sciences
- [ ] Behavioural & social sciences
- [ ] Ecological, evolutionary & environmental sciences

For a reference copy of the document with all sections, see nature.com/documents/nr-reporting-summary-flat.pdf

Life sciences study design

All studies must disclose on these points even when the disclosure is negative.

Sample size	We collected 46 NPC samples for protein expression and tumor-infiltrating immune subpopulations analysis, and collected 355 paraffin-embedded NPC samples for survival analysis.
Data exclusions	No data were excluded from the analyses.
Replication	All attempts at replications were successful.
Randomization	The samples used in this study were randomly allocated into control or experimental groups.
Blinding	The investigators were not blinded to sample allocation during experiment and outcome assessment, because results used were obtained using objective quantitative methods.

Reporting for specific materials, systems and methods

We require information from authors about some types of materials, experimental systems and methods used in many studies. Here, indicate whether each material, system or method listed is relevant to your study. If you are not sure if a list item applies to your research, read the appropriate section before selecting a response.

Materials & experimental systems

n/a	Involved in the study	
[ ]	[x]	Antibodies
[ ]	[x]	Eukaryotic cell lines
[x]	[ ]	Palaeontology and archaeology
[ ]	[x]	Animals and other organisms
[x]	[ ]	Clinical data
[x]	[ ]	Dual use research of concern

Methods

n/a	Involved in the study	
[x]	[ ]	ChIP-seq
[ ]	[x]	Flow cytometry
[x]	[ ]	MRI-based neuroimaging

Antibodies

Antibodies used	The antibodies used are listed in Supplementary Table 5.

Validation	All antibodies were validated by immune blotting, IHC or immunofluorescence imaging prior to isotope-polymer conjugation. Antibodies were tested for cell type and inter-cell location specificity within positive control tissues.

Eukaryotic cell lines

Policy information about cell lines and Sex and Gender in Research

Cell line source(s)	The human NPC cell lines (SUNE1 and HONE1) were provided and authenticated by Professor Musheng Zeng (Sun Yat-sen University Cancer Center, China). The HEK293T cell line and mouse colon cancer cell line MC38 were obtained from the American Type Tissue Culture Collection (ATCC).
Authentication	None of the cell lines were authenticated.
Mycoplasma contamination	All the cells were tested for mycoplasma contamination, and cultured for less than 2 months.
Commonly misidentified lines (See ICLAC register)	None.

Animals and other research organisms

Policy information about studies involving animals; ARRIVE guidelines recommended for reporting animal research, and Sex and Gender in Research

Laboratory animals	Six-week-old female BALB/c nude mice and C57BL/6 mice were purchased from Charles River Laboratories. A humanized NSG mouse model (Shanghai Model Organisms) was established by tail vein injection of human PBMCs (5×10^6) and validated by the detection of more than 1% human CD45+ cells in the peripheral blood of the mice one week after injection.
Wild animals	The study did not involve wild animals.
Reporting on sex	All animals used in this study were female, and no sex-based analysis was performed.
Field-collected samples	The study did not involve samples collected from the field.
Ethics oversight	Animal experiments in this study were approved by the Experimental Animal Ethics Committee, Sun Yat-sen University Cancer Center (L025501202108037) and complied with the Declaration of Helsinki.

Note that full information on the approval of the study protocol must also be provided in the manuscript.

Flow Cytometry

Plots

Confirm that:

- [x] The axis labels state the marker and fluorochrome used (e.g. CD4-FITC).
- [x] The axis scales are clearly visible. Include numbers along axes only for bottom left plot of group (a 'group' is an analysis of identical markers).
- [x] All plots are contour plots with outliers or pseudocolor plots.
- [x] A numerical value for number of cells or percentage (with statistics) is provided.

Methodology

Sample preparation	The sample preparation was described in the methods section.
Instrument	All data were obtained with a CYTOFLEX flow cytometer (Beckman Coulter).
Software	The results were analysed using Flow Jo software 10.
Cell population abundance	Minimum of 5,000 cells were counted for each analysis.
Gating strategy	The gating strategy were provided in the Supplementary Figure 9.

- [x] Tick this box to confirm that a figure exemplifying the gating strategy is provided in the Supplementary Information.
